# Exercise intolerance and rapid skeletal muscle energetic decline in human age-associated frailty

**DOI:** 10.1172/jci.insight.141246

**Published:** 2020-10-15

**Authors:** Sabra C. Lewsey, Kilian Weiss, Michael Schär, Yi Zhang, Paul A. Bottomley, T. Jake Samuel, Qian-Li Xue, Angela Steinberg, Jeremy D. Walston, Gary Gerstenblith, Robert G. Weiss

**Affiliations:** 1 Division of Cardiology, Department of Medicine, and; 2 Division of Magnetic Resonance Research, Department of Radiology, Johns Hopkins University School of Medicine, Baltimore, Maryland, USA.; 3 Philips Healthcare Germany, Hamburg, Germany.; 4 Key Laboratory for Biomedical Engineering of Ministry of Education, Department of Biomedical Engineering, College of Biomedical Engineering and Instrument Science, Zhejiang University, Hangzhou, Zhejiang, China.; 5 Divison of Geriatric Medicine and Gerontology, Department of Medicine, Johns Hopkins University School of Medicine, Baltimore, Maryland, USA.

**Keywords:** Aging, Bioenergetics, Mitochondria

## Abstract

**BACKGROUND:**

Physical frailty in older individuals is characterized by subjective symptoms of fatigue and exercise intolerance (EI). Objective abnormalities in skeletal muscle (SM) mitochondrial high-energy phosphate (HEP) metabolism contribute to EI in inherited myopathies; however, their presence or link to EI in the frail older adult is unknown.

**METHODS:**

Here, we studied 3 groups of ambulatory, community-dwelling adults with no history of significant coronary disease: frail older (FO) individuals (81 ± 2.7 years, mean ± SEM), nonfrail older (NFO) individuals (79 ± 2.0 years), and healthy middle-aged individuals, who served as controls (CONT, 51 ± 2.1 years). Lower extremity SM HEP levels and mitochondrial function were measured with ^31^P magnetic resonance (MR) techniques during graded multistage plantar flexion exercise (PFE). EI was quantified by a 6-minute walk (6MW) and peak oxygen consumption during cardiopulmonary testing (peak VO_2_).

**RESULTS:**

During graded exercise, FO, NFO, and CONT individuals all fatigued at similar SM HEP levels, as measured by ^31^P-MR. However, FO individuals fatigued fastest, with several-fold higher rates of PFE-induced HEP decline that correlated closely with shorter exercise duration in the MR scanner and with 6MW distance and lower peak oxygen consumption on cardiopulmonary testing (*P* < 0.001 for all). SM mitochondrial oxidative capacity was lower in older individuals and correlated with rapid HEP decline but less closely with EI.

**CONCLUSION:**

Several-fold faster SM energetic decline during exercise occurs in FO individuals and correlates closely with multiple measures of EI. Rapid energetic decline represents an objective, functional measure of SM metabolic changes and a potential new target for mitigating frailty-associated physical limitations.

**FUNDING:**

This work was supported by NIH R21 AG045634, R01 AG063661, R01 HL61912, the Johns Hopkins University Claude D. Pepper Older Americans Independence Center P30AG021334, and the Clarence Doodeman Endowment in Cardiology at Johns Hopkins.

## Abstract 2

Rapid exercise-induced skeletal muscle high-energy phosphate decline occurs in frail, older individuals and is closely linked to exercise intolerance and fatigue.

## Graphical abstract

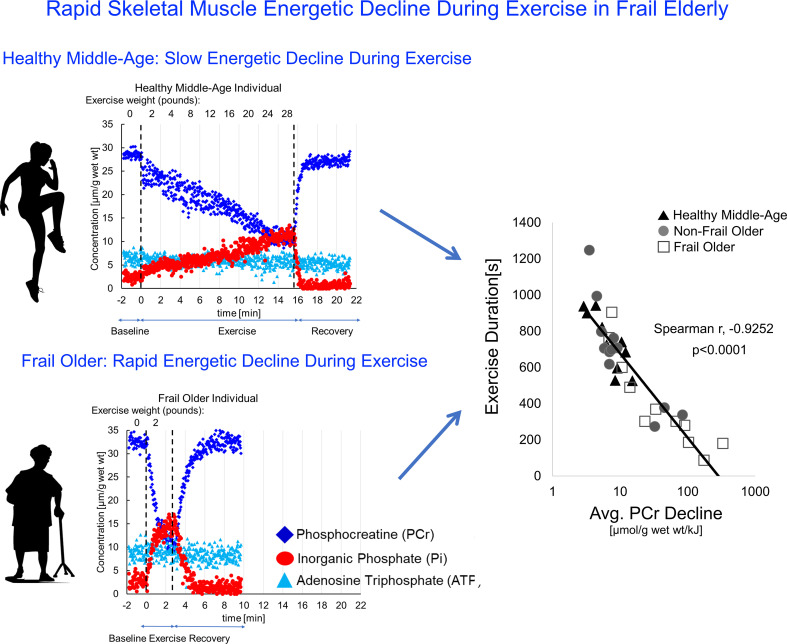

## Introduction

Physical frailty in older adults is characterized by self-reported fatigue, weakness, slowness, low activity, and unintentional weight loss (1). Approximately 15% of adults in the United States over the age of 65 years are frail and at increased risk of falls, disability, hospitalization, loss of independence, and cardiovascular death (2, 3). The factors underlying the fatigue and exercise intolerance (EI) accompanying physical frailty, however, remain poorly understood.

Fatigue, frailty, and EI are related but distinct terms with relevance to aging populations. Fatigue, often defined as the subjective sense of tiredness, is a core criterion of the physical frailty phenotype (1) and commonly reported among older individuals, especially among those who have physical disabilities, limited mobility, or difficulty with daily activities, even in the absence of frailty (4, 5). However, the sense of tiredness does not specify the duration or intensity of activity that caused the fatigue. Fatigability, or the symptom of fatigue normalized to the inciting activity duration and intensity, better characterizes this prognostic symptom in older individuals (4, 6–8). Likewise, EI is often defined as an impairment in objective measures of volitional activity or exercise performance.

Age-associated changes in skeletal muscle (SM) may contribute to the unique entities of fatigue, EI, and frailty. To the extent that normal aging is characterized by a decline in biologic systems responsible for maintaining homeostasis in response to physiologic stress, the decline in SM typically manifests as an age-related fiber atrophy, reduced muscle mass and strength or sarcopenia (9–12), oxidative damage to mitochondrial DNA, and reduced mitochondrial density in some studies (11–14). Such age-associated mitochondrial changes could, in theory, contribute to these geriatric functional syndromes.

Muscle exhaustion during exercise is often considered an energy-deficient state because isolated SM preparations at performance failure have reduced high-energy phosphates (HEPs) (i.e., phosphocreatine [PCr] and ATP) and free energy release from ATP hydrolysis (ΔG_~ATP_), as well as an accumulation of products of ATP degradation (i.e., inorganic phosphate [Pi] and H^+^)(7, 8, 15–17). Impaired SM energy metabolism contributes to EI in people with skeletal myopathies (18, 19), and recent data suggest both an age-related increase in the ATP cost of SM contraction and a role of impaired muscle bioenergetics in poorer (or slower) age-related walking speed performance (20, 21). However, the extent to which energetic abnormalities and mitochondrial dysfunction are linked to age-associated fatigability and EI in older individuals with the frailty phenotype is not presently known.

Assessment of the potential role of impaired SM energetics in fatigability in frail older (FO) individuals requires evaluation of SM energetic parameters at rest, during exercise performed to performance fatigue, and during recovery. Especially important biochemically are measures of absolute HEP concentrations (rather than ratios), rates of ATP synthesis, and rates of change of these energetic parameters during exercise and recovery. Ideally, exercise would include a common range of exercises that can be performed by nonfrail older (NFO) and FO individuals and would be derived from a small muscle group so as to capture intrinsic skeletal muscle metabolic abnormalities and thereby diminish the impact of central hemodynamic and cardiac factors.

Here, we set out to test the hypothesis that SM mitochondrial oxidative capacity is reduced and that HEP decline during exercise is faster in FO individuals than in middle-aged or age-matched, NFO individuals. We used an energetic plantar flexion exercise (PFE) fatigability test with concomitant noninvasive in vivo phosphorus magnetic resonance spectroscopy (^31^P-MRS) measures of SM HEPs, Pi, intracellular pH, and mitochondrial maximal oxidative capacity. Three groups were studied, including community-dwelling NFO individuals, older individuals with a frailty clinical phenotype (FO individuals), and healthy middle-aged participants who served as controls (CONT). Finally, to determine the real-world relevance of energetic observations, we related the energetic and EI exhibited in the MRI scanner with walking and whole-body exercise capacity. These findings are consistent with the hypothesis that reduced SM energy metabolism is closely related to EI in age-associated frailty, and that an energetic myopathy contributes to the age-associated physical frailty phenotype.

## Results

### Patient characteristics.

There were no differences in age or BMI between the FO and NFO groups, although both older groups had higher BMI than the middle-aged CONT group (Table 1). As expected, objective EI was most marked in FO individuals as shown by the shortest 6-minute walk distance (6MW), whereas 6MW was shorter in the NFO individuals than in the CONT group (Table 1, *P* < 0.01). Respiratory exchange ratios (RERs) were similar among the groups and indicated adequate effort during cardiopulmonary exercise testing (CPET). Likewise, FO individuals had significantly lower bicycle peak oxygen consumption (peak VO_2_) compared with both the controls and NFO individuals (Table 1, *P* < 0.01).

### PFE performance and test of global function.

**Figure 1:**
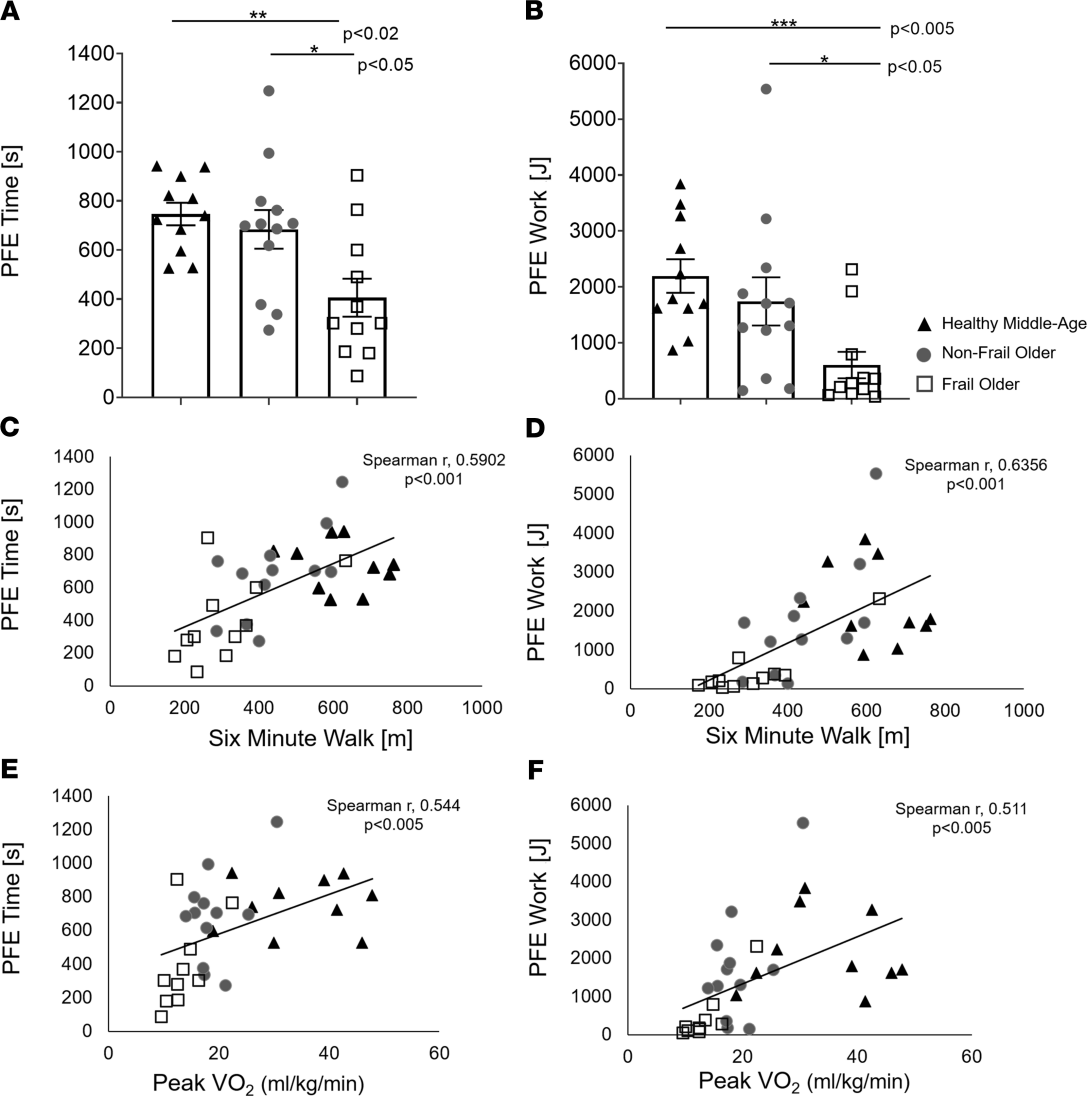
Plantar flexion exercise performance and tests of global performance. (**A**) Plantar flexion exercise (PFE) time, (**B**) total PFE work, and (**C–F**) correlations between indices of PFE performance (PFE time and PFE work) and established global functional indices (6MW and peak VO_2_ at CPET). Control (CONT, *n* = 11, black triangles), nonfrail older (NFO, *n* = 12, dark-gray circles), and frail older (FO, *n* = 11, open squares) individuals. Data are individual points and shown as mean ± SEM. (**A**) ANOVA with multiple comparisons tests, (**B**) Kruskal-Wallis with Mann-Whitney *U* tests, and (**C–F**) Spearman’s correlations were used. *P < 0.05, **P < 0.02, ***P < 0.005, ****P < 0.001.

FO individuals had the most profound EI during graded PFE performed in the MRI as shown by the shortest mean exercise time and lowest total work performed in comparison with both the CONT and NFO individuals (Figure 1, A and B). Moreover, FO individuals performed only one-third of the total work performed by their NFO counterparts. Importantly, metrics of PFE performance, including exercise duration and total work, significantly correlated with established measures of global functional capacity, including 6MW and bicycle peak VO_2_ (Figure 1, C–F). As expected, FO had the poorest performance inside and outside of the MRI. Notably, however, PFE performance in the MRI scanner paralleled conventional global functional assessments in this cohort.

### SM energetics at rest and at fatigue.

**Figure 2:**
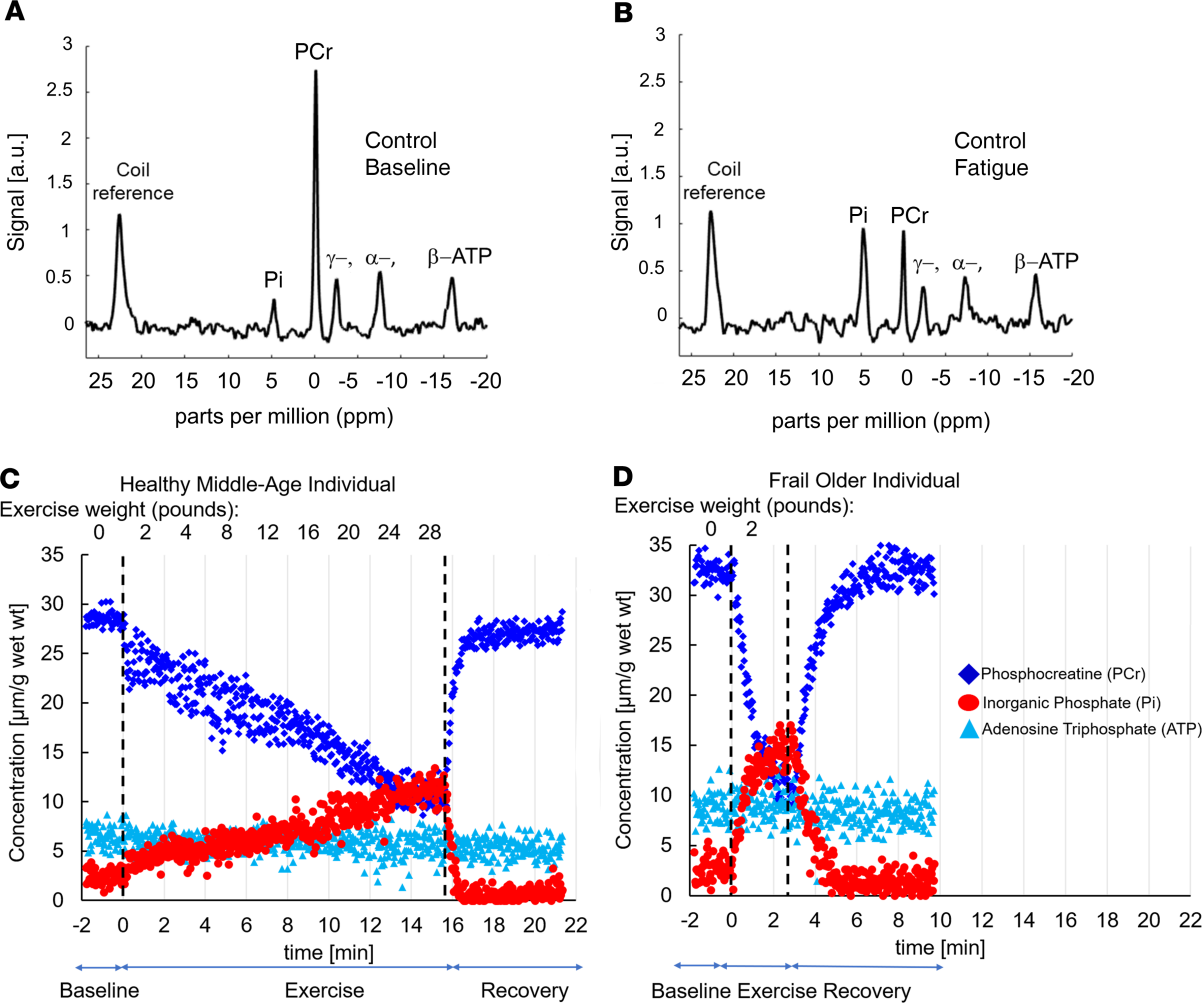
Dynamic measures of skeletal muscle high-energy phosphates. Representative ^31^P-MR spectra at baseline (**A**) and at fatigue (**B**) of a healthy middle-aged subject. Time course of skeletal muscle high-energy phosphate metabolites: PCr (dark blue) and ATP (light blue) and inorganic phosphate (red) before, during, and after exercise in a healthy middle-aged (**C**) and frail older subject (**D**). The rate of PCr decline during exercise is several-fold faster in the frail older subject.

**Figure 3:**
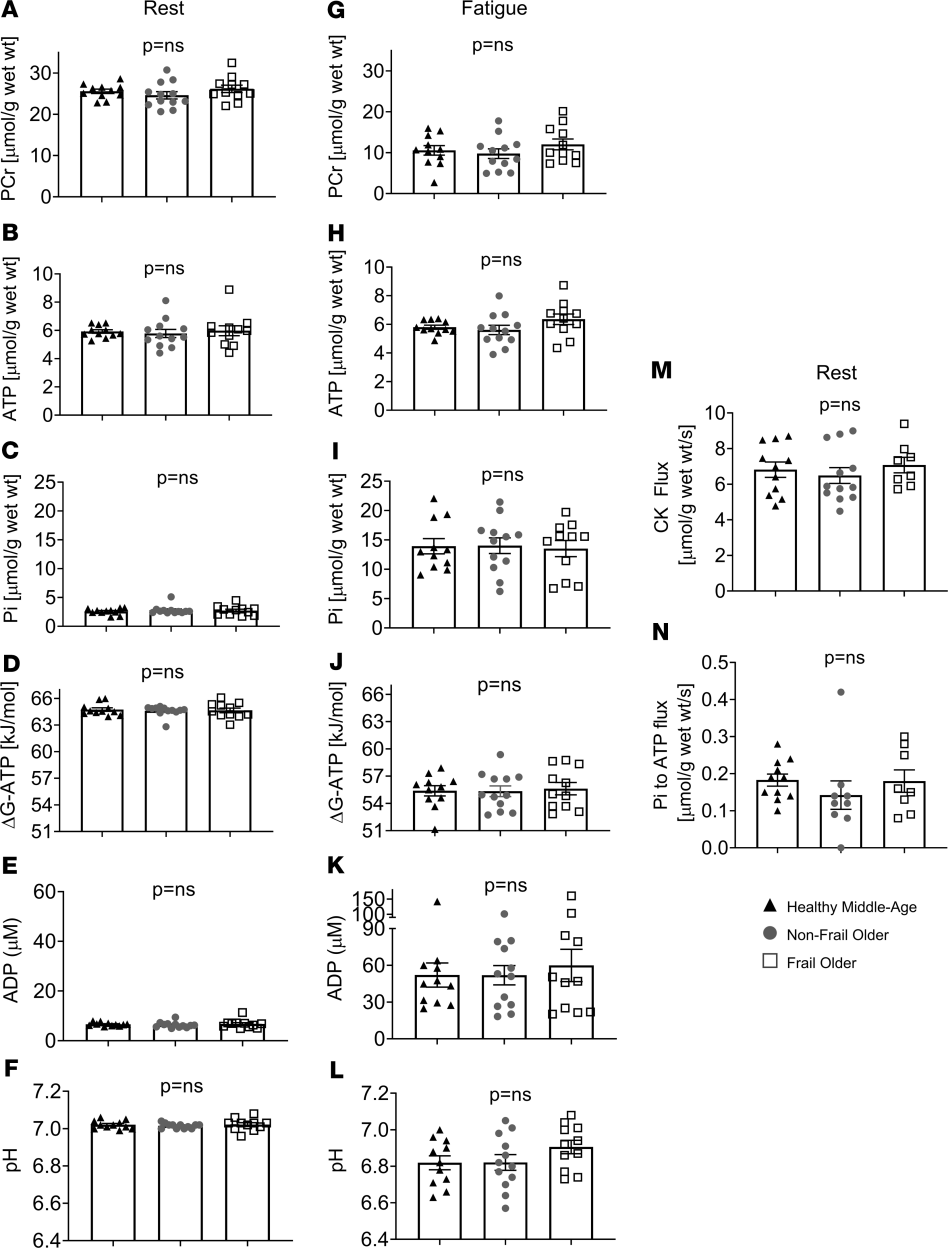
Energetic parameters at rest and at fatigue. Skeletal muscle energetic parameters (PCr, ATP, Pi, ΔG_~ATP_, ADP, pH) during resting conditions (**A–F**) and at performance fatigue (**G–L**). There were no significant differences in any of these metabolic parameters at rest or at performance fatigue among the 3 groups. CONT (*n* = 11, black triangles), NFO (*n* = 12, dark-gray circles), FO (*n* = 11, open squares). Skeletal muscle unidirectional ATP synthesis rates from PCr through CK at rest (**M**, CONT, *n* = 11, black triangles; NFO, *n* = 12, dark-gray circles; FO, *n* = 8, open squares) and of ATP synthesis rates from Pi at rest (**N**, CONT, *n* = 11; black triangles, NFO, *n* = 9; dark-gray circles, FO, *n* = 8, open squares) did not differ among the 3 groups. Data are individual data points and shown as mean ± SEM. ANOVA and Kruskal-Wallis ANOVA were used.

Figure 2 shows representative ^31^P-MRS spectra and the time course of energetic data for 2 individuals. SM metabolite concentrations, including PCr and ATP, were similar under resting conditions in all 3 groups (Figures 2 and 3). Additionally, ATP synthesis rates did not differ significantly among the 3 groups at rest (Figure 3). Dynamic Pi started at similarly low concentrations at rest and accumulated during PFE to equivalent concentrations at fatigue among the 3 groups, with a similar intracellular acidosis among all 3 cohorts (Figures 2 and 3). Likewise, there was a progressive, parallel decline in PCr during PFE with preservation of ATP in all 3 cohorts. Notably, at fatigue, all 3 groups had similar reductions in mean PCr, intracellular pH, and ΔG_~ATP_, along with similar Pi and ADP accumulation (Figure 3). Thus, CONT, NFO, and FO individuals had a similar SM energetic profile, reflecting a similar SM resting reserve and common energetic limit at performance fatigue, in spite of differences in exercise duration and the total work performed.

### Rate of SM HEP depletion during exercise and functional capacity.

**Figure 4:**
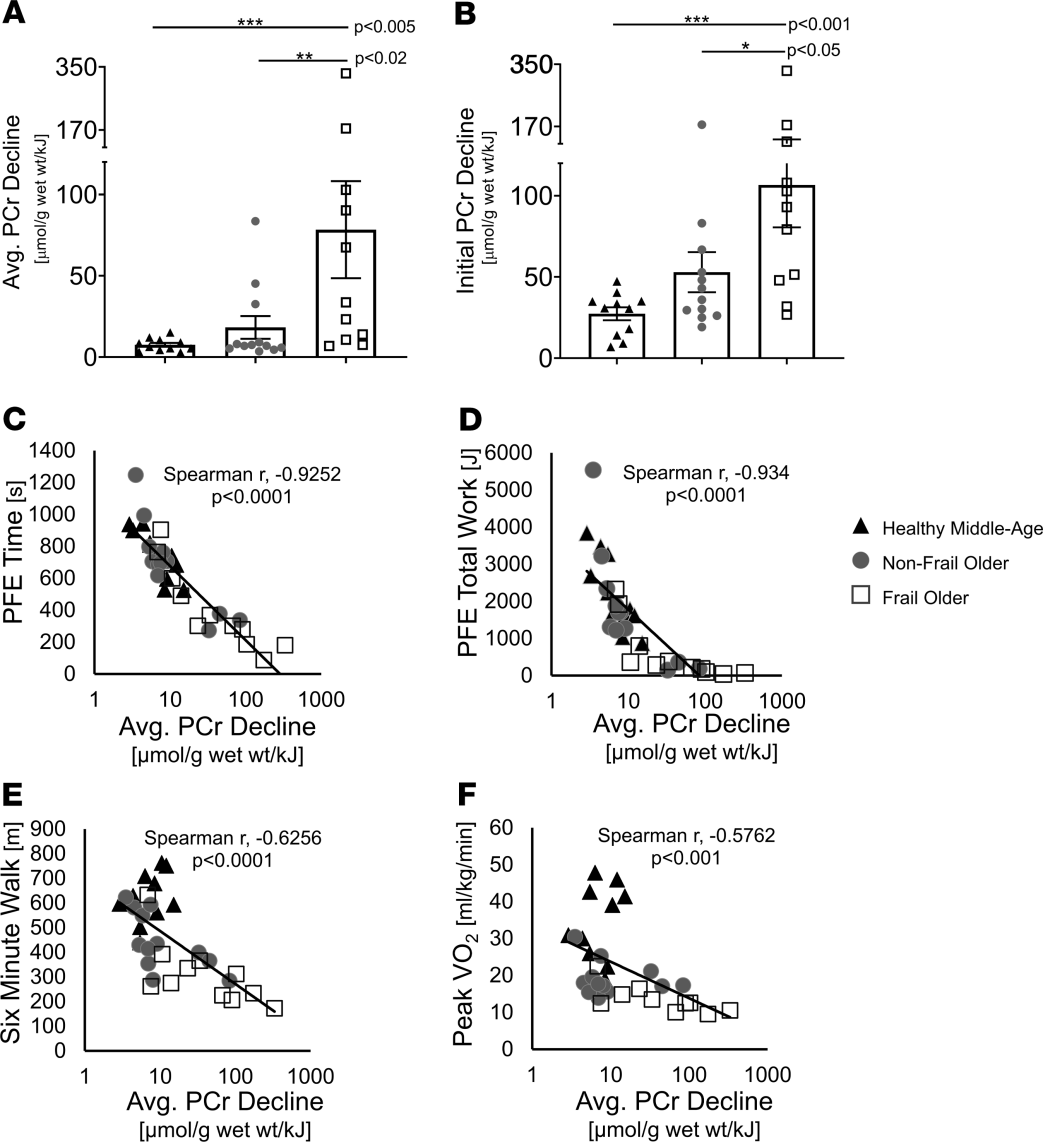
Rapid exercise-induced energetic decline and functional capacity. Both the average rate of PCr decline during all of PFE (**A**) and the initial rate of PCr decline during the first 4 minutes of PFE (**B**) were significantly faster in frail older individuals (open squares) than in nonfrail older (dark-gray circles), and healthy middle-aged participants (black triangles). (**C**) Short exercise time was strongly associated with rapid energetic decline in that there was an inverse correlation between PFE time and the rate of PCr decline (*P* < 0.0001). Total work performed during PFE (**D**), 6-minute walk distance (**E**), and peak VO_2_ (**F**), all correlated inversely and significantly with the rate of PCr decline during exercise. CONT (*n* = 11, black triangles), NFO (*n* = 12, dark-gray circles), FO (*n* = 11, open squares). Data are individuals points and shown as mean ± SEM. Kruskal-Wallis ANOVA with Mann-Whitney *U* tests and Spearman’s correlations were used. *P < 0.05, **P < 0.02, ***P < 0.005, ****P < 0.001.

Given that FO individuals fatigue faster (Figure 1) but with similar HEP concentrations at fatigue (Figure 3), the mean rate (Figure 3) of phosphocreatine depletion during exercise normalized to the work of activity performed was 10-fold higher in FO individuals than the mean rate in CONT individuals and 4-fold higher than the mean rate observed in age-matched NFO individuals (FO, 78.4 ± 29.9 μmol/g/kJ; NFO, 18.2 ± 7.0 μmol/g/kJ, *P <* 0.02; CONT, 7.5 ± 1.2 μmol/g/kJ, *P <* 0.005; Figure 4A). The average rate of PCr decline was also determined during the initial stages of low-intensity exercise performed by nearly all participants to directly compare groups at the same workloads and to minimize differences in cardiac output among the 3 groups that may be present at peak exercise. If peak cardiac output is limiting, it would impact rates averaged over the entire exercise interval. The initial rate of PCr decline in the first 4 minutes was still fastest in FO individuals compared with that in CONT (*P* < 0.001) and NFO individuals (*P* < 0.05; Figure 4B). Even when not normalized to work performed, FO individuals still had several-fold faster rates of energetic depletion during the first stage of exercise when all subjects lifted the same weight (CONT, *P* < 0.005; NFO, *P* < 0.05; Supplemental Figure 9; supplemental material available online with this article; https://doi.org/10.1172/jci.insight.141246DS1)**.**

There was a strong inverse correlation between the rate of PCr decline during exercise and PFE duration (Spearman’s correlation coefficient, *r* = [–] 0.9252, *P* < 0.0001; Figure 4C) as well as total PFE work performed (*r* = [–] 0.934, *P* < 0.0001; Figure 4D). In addition, the rate of PCr decline during PFE in the MRI correlated inversely with 6MW performance (*r* = [–] 0.6256, *P* < 0.0001; Figure 4E), and peak VO_2_ at CPET (*r* = [–] 0.5762, *P* < 0.001; Figure 4F). These findings indicate that the rate of SM HEP depletion was several-fold faster in FO than in NFO and CONT individuals, the rapid depletion in FO began early during even low-level exercise, and that the rates of HEP decline correlated in all with diminished performance during PFE in the MRI scanner and with reduced established functional parameters measured outside of the MRI scanner. These observations are consistent with the premise that rapid SM energy depletion contributes to EI in FO individuals.

### Maximal mitochondrial oxidative and functional capacity.

**Figure 5:**
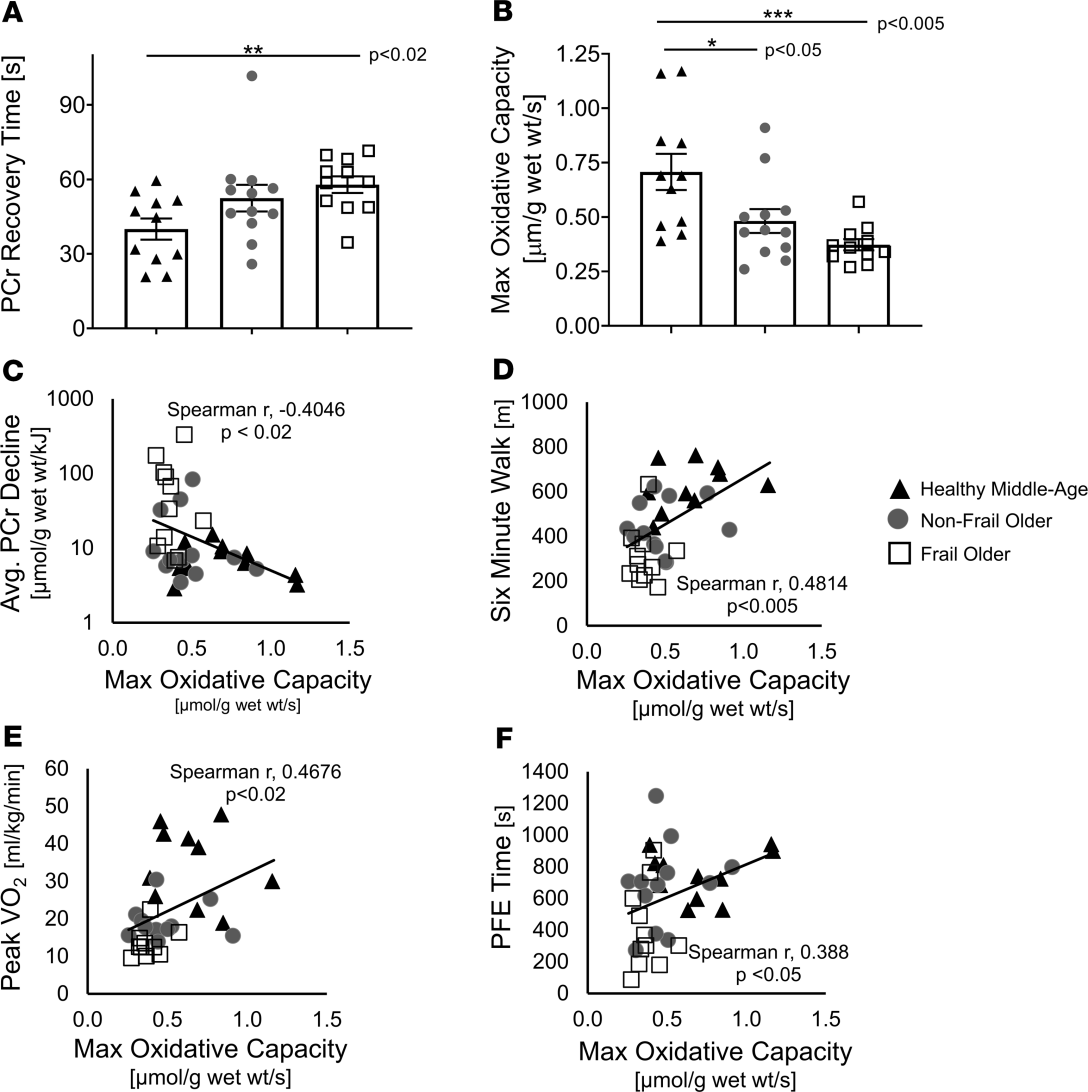
Mitochondrial capacity and functional performance. Recovery time of creatine phosphate (PCr) after plantar flexion exercise (PFE) is longer (**A**), and maximum oxidative capacity is reduced (**B**) in frail older individuals. The rate of PCr decline during PFE correlates with reduced maximum oxidative capacity (**C**, *P* < 0.02). Correlations between 6MW and maximum oxidative capacity (**D**), between peak VO_2_ and maximum oxidative capacity (**E**), and between PFE time and maximum oxidative capacity (**F**). Data are individual points and shown as mean ± SEM. CONT (*n* = 11, black triangles), NFO (*n* = 12, dark-gray circles), FO (*n* = 11, open squares). (**A**) Kruskal-Wallis with Mann-Whitney *U* tests, (**B**) ANOVA with multiple comparisons tests, and (**C–F**) Spearman’s correlations were used. *P < 0.05, **P < 0.02, ***P < 0.005, ****P < 0.001.

Rapid HEP decline during exercise could be due to increased ATP consumption and/or to decreased mitochondrial ATP production. Therefore, to evaluate the latter, maximal mitochondrial oxidative capacity was measured and FO individuals had longer average post-exercise PCr recovery times (FO, 57.9 ± 3.4 seconds; CONT, 40 ± 4.2 seconds; *P* < 0.02; Figure 5A) and nearly one-half of the maximal oxidative capacity of CONT (FO, 0.37 ± 0.03 μmol/g/s; CONT, 0.71 ± 0.08 μmol/g/s, *P* < 0.005; Figure 5B). NFO individuals had an intermediate PCr recovery time (NFO, 52.5 ± 5.4 seconds) and oxidative capacity (NFO, 0.48 ± 0.05 μmol/g/s; Figure 5, A and B) that differed significantly from CONT, but not from FO. Collectively, maximal oxidative capacity inversely correlated with the logarithm of the rate of PCr decline (*r* = –0.4046, *P* < 0.02; Figure 5C). The maximal oxidative capacity was directly correlated with the functional parameters of PFE time (*P* < 0.05) in the MRI, with 6MW (*P* < 0.005), and with peak VO_2_ (*P* < 0.02; Figure 5, D–F) with the caveats that the range of oxidative capacities was reduced in FO versus CONT and the relationships were not as tight as those with the rate of PCr decline (Figure 4C vs. Figure 5F). Rapid PCr decline during exercise was inversely associated with mitochondrial function (Figure 5C). Thus, mitochondrial function was significantly reduced in older individuals, trended lowest in FO individuals, and correlated inversely with rapid energetic decline during exercise.

### SM mass and intramuscular fatty replacement.

**Figure 6:**
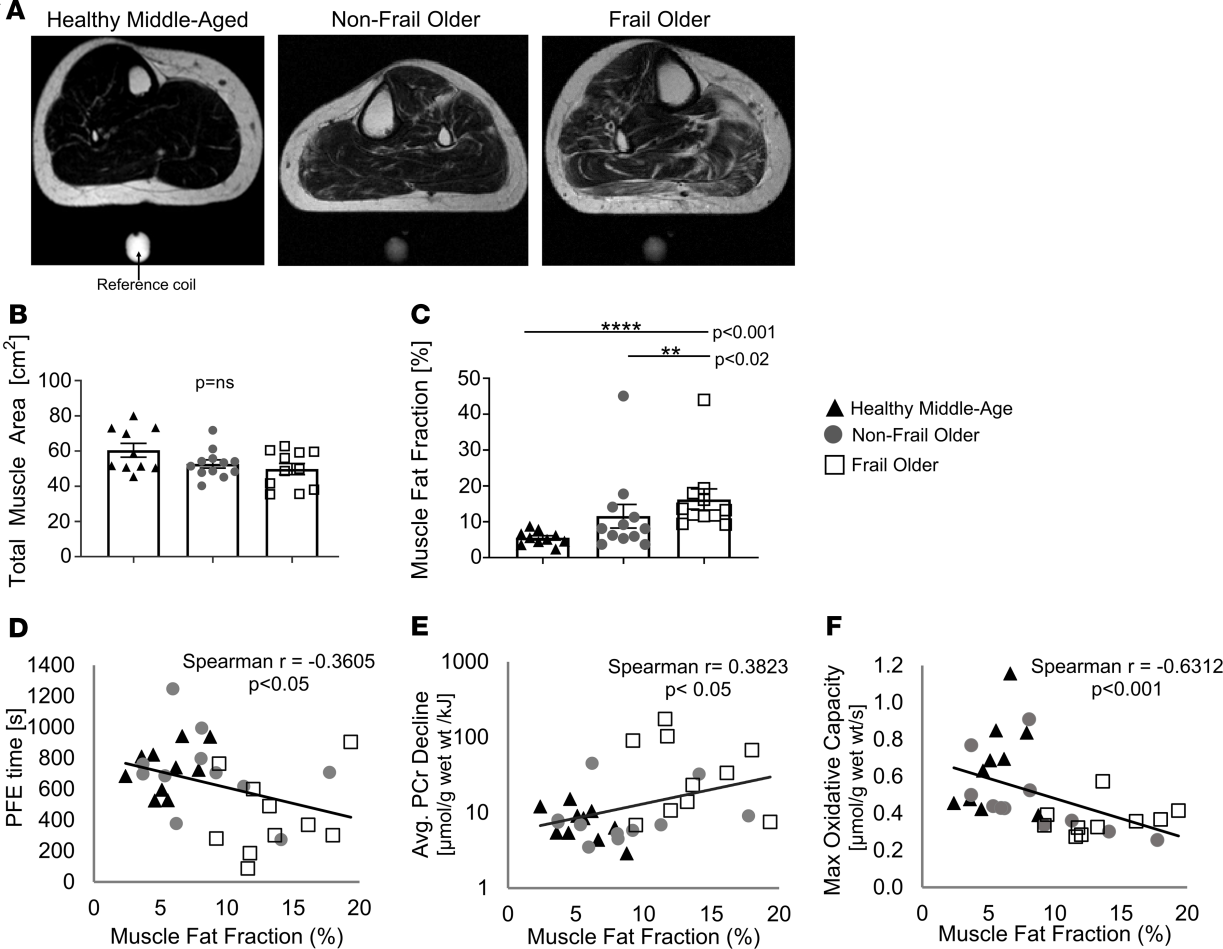
Muscle fat, energetics, and exercise duration. (**A**) Magnetic resonance images showing fat distribution (white signal) in the calf of a healthy middle-aged (left), nonfrail older (middle), and frail older (right) individual. Total cross-sectional muscle areas across the 3 groups did not differ (**B**), however muscle fat content was significantly elevated in frail older individuals (**C**). (**D**) Correlation between PFE time and muscle fat fraction percent (without outliers). (**E**) Correlation between average PCr decline and muscle fat fraction percent (without outliers). (**F**) Correlation between maximal oxidative capacity and muscle fat fraction percent (without outliers). See Supplemental Data for all comparison figures with and without outliers. Muscle fat fraction was considered an outlier if intramuscular fat content exceeded 42%, which included 2 participants (1 frail, 1 nonfrail). Data are individual points and shown as mean ± SEM. CONT (*n* = 10 black triangles), NFO (*n* = 11 dark-gray circles), FO (*n* = 10 open squares). Spearman’s correlations were used. A *P* value less than 0.05 was considered significant with or without outliers. *P < 0.05, **P < 0.02, ***P < 0.005, ****P < 0.001.

To determine whether sarcopenia accounts for the PFE intolerance observed in this FO cohort, we measured muscle cross-sectional area by MRI and there was no difference among the 3 groups (Figure 6, A and B). Because muscle lipid accumulation occurs in frail and prefrail individuals (22), we determined whether SM lipid accumulation occurs with frailty, and whether it is associated with the observed energetic and functional changes. FO individuals had 3 times the mean intramuscular fat fraction of CONT (FO, 16.2% ± 2.9%; CONT, 5.5% ± 0.6%, *P* < 0.001; Figure 6C). NFO individuals exhibited intermediate fat fraction (NFO, 11.5% ± 3.3%; Figure 6C), which was not significantly different from CONT (*P* = 0.0503, NS), but significantly less than in FO individuals (*P* < 0.02). There was a direct correlation between muscle fat fraction and BMI (Supplemental Figure 1), and an inverse correlation between muscle fat fraction and PFE time, even with the highest fat fraction outliers omitted (Figure 6D and Supplemental Figure 2). However, BMI did not correlate with exercise tolerance during PFE (Supplemental Figure 3). Muscle fat fraction directly correlated with average PCr decline (Spearman’s correlation coefficient, *r* = 0.4759, *P* < 0.02; Figure 6E and Supplemental Figure 4) and was inversely correlated with maximal oxidative capacity (Spearman’s correlation coefficient, *r* = –0.5185, *P* < 0.02; Figure 6F and Supplemental Figures 5 and 7), even with outliers omitted. Thus, FO individuals with the highest intramuscular fat exhibited the most rapid rates of PCr decline and the most impaired mitochondrial function.

## Discussion

Although the physical frailty phenotype in older individuals is an independent predictor of disability, hospitalizations, loss of independence, and cardiovascular death (1, 3), the factors underlying this vulnerability and the accompanying fatigue and EI in this population are incompletely understood. This study used an energetic ^31^P-MRS fatigability test in FO and nonfrail individuals with a range of exercise tolerances to provide insight into the hypothesis that SM mitochondrial and energetic abnormalities occur with aging, accelerate with frailty, and are closely related to EI, slowness, and increased fatigability. Four novel findings are reported in this study. First, SM HEP stores do not differ at rest or at performance fatigue in FO or NFO individuals as compared with CONT individuals, consistent with a common energetic limit for fatigue regardless of frailty status and age. Second, the mean rate of SM HEP decline during exercise was 4- to 10-fold faster in FO than in NFO and CONT individuals, respectively, and was closely associated with EI exhibited both during PFE in the MRI scanner and with walking and whole-body exercise capacity. Third, the rapid SM HEP decline during exercise in FO individuals cannot be explained by a reduction in the primary muscle phosphagen reaction, creatine kinase. However, rapid SM HEP depletion was inversely related to maximal mitochondrial oxidative capacity among all study subjects, and was reduced by nearly 50 percent in older as compared with middle-age individuals. Fourth, SM fat content increased several-fold in FO individuals and was associated with rapid energetic decline during exercise, shortened exercise duration, and reduced mitochondrial oxidative capacity. These observations are consistent with the hypothesis that EI in FO individuals is closely related to rapid SM energetic decline during exercise, reduced mitochondrial energy metabolism with aging, and marked SM fatty infiltration, and that these findings may occur in some frail individuals in the absence of muscle atrophy.

In isolated preparations of SM, fatigue is related to HEP depletion, reduced Gibbs free energy release from ATP hydrolysis (ΔG_~ATP_), and the accumulation of Pi and H^+^ from ATP degradation (7, 16, 17, 23). Therefore, decreased SM energy metabolism is a plausible contributor to fatigability in age-associated frailty since normal muscle function is inherently dependent on ATP and bioavailable energetic stores (7, 24). In fact, a precedent exists for SM energy deprivation and impaired ATP metabolism to occur and contribute to muscle fatigue and weakness in muscular dystrophies and metabolic myopathies (18, 19, 25–27). An animal model of age-associated frailty, the aged homozygous IL-10–knockout mouse, has in vivo SM reductions at rest in HEPs, ATP synthesis rates, and ΔG_~ATP_ (28). This is the first report to our knowledge to directly measure absolute concentrations of SM ATP and HEPs in older individuals with frailty at rest and during exercise-induced energy decline performed to fatigue. Our study does not depend on the assumption that ATP concentrations are constant across age groups.

We observed that FO individuals experience subjective fatigue at lower workloads and at shorter exercise times but at the same SM concentrations of PCr, Pi, and ATP as well as pH, as NFO and CONT individuals (Figures 3 and 4). The observation that performance fatigue occurs at a common energetic level in all individuals studied does not prove, but is consistent with, the hypothesis that SM energetic depletion and/or catabolite accumulation is a determinate of fatigue. If nonenergetic factors primarily determined the time of fatigue, then performance fatigue could occur at variable HEP concentrations. This was not observed. This common energetic limit coupled with the observation that HEPs decline faster in frail individuals with high fatigability (i.e., reach fatigue at lower workloads and less activity duration), indicates that performance fatigue in frail individuals is very closely associated with more rapid SM HEP depletion (Figure 4C).

The faster SM energetic decline observed in FO people at matched workloads (Figure 4B) could be due to reduced HEP production and/or increased HEP consumption. Energetic decline during exercise is buffered by muscle phosphagen transfer reactions such as the creatine kinase reaction, which serves as the primary muscle energy reservoir during exercise by rapidly and reversibly converting PCr and ADP to creatine and ATP (29). Activities of daily living that can be affected by the frailty phenotype include standing, initiation of walking, and grip strength; all processes that use ATP from phosphagen reactions and strongly predict disability in older populations (30–35). An in vivo study of age-related frailty in mice showed reduced SM ATP flux through creatine kinase at rest (28) and lower SM creatine kinase activity with aging (36). However, our finding that in vivo SM ATP synthesis from creatine kinase is not reduced with frailty indicates that reduced SM creatine kinase is not the cause of EI in these older frail individuals.

Unlike phosphagen reactions, which can provide ATP during brief episodes for burst activities, most sustained muscle ATP generation occurs in the mitochondria by oxidative phosphorylation (11). Mitochondrial function can directly impact SM performance, and deleterious oxidative damage to mitochondrial DNA is thought to decrease mitochondrial content and function with increasing age (11, 37, 38). Previous studies have shown that aerobic capacity is lower in older inactive individuals than in comparison with their younger inactive counterparts (12), and mitochondrial-coupling efficiency is also lower in older than in younger adults (39). Furthermore, older individuals with higher fatigability on treadmill exercise tests have lower capacity for oxidative phosphorylation than older individuals with lower fatigability (40). Lower mitochondrial capacity is also associated with slower walking speeds and lower muscle strength in older individuals (41, 42). Other studies suggest that mitochondrial density and function in older individuals are related to their activity level (43, 44), and that mitochondrial function is associated with walking performance in active older adults, but not in sedentary older adults (45). Chronic exercise training, and thus increased physical activity, may modify mitochondrial dysfunction (46, 47). Furthermore, SM phosphocreatine recovery is delayed and mitochondrial respiratory complex protein and activity reduced in older prefrail individuals as compared with active older adults (48). These prior studies are consistent with the hypothesis that impaired mitochondrial function occurs in older individuals and is associated with reduced functional capacity and lower muscle strength. We report here that SM maximal oxidative capacity is reduced in older individuals, trends lower in those with frailty, and is linked to rapid HEP decline and EI in older individuals. However, another novel observation here is that the relationship between EI and rapid energetic decline (Figure 4C) exists and is much stronger than the relationship between EI and reduced mitochondrial function (Figure 5F).

We observed a 3-fold increase in intramuscular fat in FO individuals as compared with that of CONT subjects (*P* < 0.001), and 30 percent more fat in comparison with age-matched NFO (*P* < 0.02) individuals. This is consistent with data previously reported in which FO individuals were noted to have increased intramuscular adipose tissue by thigh MRI in comparison with nonfrail, age- and BMI-matched peers (49). In addition, muscle IL-6 mRNA and protein content are increased in frail individuals and a significant association exists between intramuscular fat and IL-6 mRNA and IL-6 protein expression (49). In addition to confirming increased muscle fat in FO individuals, for the first time to our knowledge this study shows that muscle fat accumulation and rapid energetic decline are closely associated and, in turn, closely linked to EI and increased fatigability. Furthermore, this work demonstrates that SM maximal oxidative capacity is inversely associated with intramuscular fat content. Taken together, all of these observations are consistent with a working metabolic framework of age-related frailty in which the SM exhibits normal HEP stores at rest, but very rapid energetic depletion to a common energetic limit at fatigue that is closely associated with profound EI and increased fatigability. The rapid SM energetic decline is associated with significantly reduced mitochondrial oxidative capacity and markedly increased muscle fat accumulation. Although our data do not answer the question of whether mitochondrial abnormalities cause fatty accumulation or vice versa, they suggest that fatty replacement of SM and the previously described proinflammatory state (50) contribute to impaired SM HEP metabolism possibly through paracrine effects (51–53) and, in turn, increased fatigability in age-related frailty.

### Limitations.

This study recruited a relatively small number of subjects. Additionally, we did not study individuals with the most extreme clinical manifestations of frailty since ambulation was required for participation in our protocol. Despite the modest sample size and absence of individuals with extreme manifestations of frailty, the study was sufficient to detect highly significant SM energetic abnormalities and relationships between energetic abnormalities and reduced functional performance. Currently, this study is unable to ascribe the faster rate of HEP depletion exclusively to impaired mitochondrial function in the frail subjects. For example, we did not study the extent to which abnormalities in macro- or microcirculatory oxygen delivery with aging (54, 55) could impair mitochondrial function during exercise and contribute to the rapid exercise-induced energetic decline. We were not able to obtain dynamic lower extremity macrocirculatory blood flow measurements during exercise because of significant artifacts and limitations of MRI without contrast agent administration. However, we did not observe a significant difference in resting peak blood flow between FO and NFO individuals (Supplemental Figure 6). Thus, although PFE involves relatively small muscle groups not likely limited by peak cardiac output, especially during early low-level exercise, additional studies are needed to probe the possibility that abnormal blood flow, dynamic shunting with exercise, or microcirculatory abnormalities contribute to the rapid energetic decline in FO individuals. This study investigated only the muscles responsible for plantar flexion although prior work suggests that age-associated changes in mitochondrial capacity differ among muscle groups (43). We believe it would be important to study other muscle groups in the future but emphasize that plantar flexion is important for ambulation and several activities of daily living, and that these findings correlated both with 6MW and whole-body peak VO_2_ during bicycle exercise as well as walking speed (Supplemental Figure 8). We also recognize that catabolite accumulation (Pi and H^+^) could contribute significantly to exercise-induced SM fatigue in addition to rapid HEP decline. Therefore, although one interpretation of the rapid energetic decline with a common energetic limit is that FO individuals “run out of fuel faster” and that is closely related to them stopping exercise earlier, another is that FO individuals accumulate catabolites faster, which causes them to stop earlier. Because high-energy metabolite decline and catabolite accumulation are intimately linked in vivo, in vitro systems would likely have to be explored in the future to distinguish their relative contribution to SM fatigue. Although tissue biopsies could provide more molecular insights and would have been of interest, they were not obtained because of their invasive nature and reluctance of many older subjects to undergo a surgical procedure. The ^31^P-MR detection of rapid SM energetic decline during exercise offers an objective, noninvasive metric for quantifying metabolic changes associated with physical frailty and may be used in the future to test metabolic treatment strategies. However, additional studies are needed to determine whether these metabolic abnormalities are themselves related to increased risk of disability and reduced resilience, in which case, they may eventually complement the well-established but partly subjective frailty criteria.

### Conclusions.

Physically FO individuals have profound EI but similar SM energy metabolites at rest as NFO individuals, both in HEP concentrations and ATP synthesis rates via the creatine kinase reaction (i.e., they begin exercise with equivalent energetic fuel). Frail individuals, however, exhibit rapid depletion of HEPs, which decline to a level common to that of NFO and CONT individuals during exercise, and the rapid rate of energy decline correlates closely with objective metrics of EI and decreased global functional capacity. Maximal mitochondrial oxidative capacity is reduced in older individuals and is associated with rapid energetic decline and exercise performance. In addition, intramuscular fat content is increased in FO individuals and associated with rapid exercise-induced energetic decline. Although physical frailty in the aging population is prevalent and an independent predictor of falls, morbidity, and cardiovascular mortality, its underlying factors are poorly understood. These observations identify rapid, exercise-induced SM energetic decline in frailty and its close relation to decreased physical function and ambulation. Our study supports recognition of frailty, at least in part, as a SM metabolic myopathy of aging, offers a noninvasive energetic fatigability test to objectively quantify these changes, and suggests SM energetic abnormalities as new therapeutic targets to possibly mitigate this debilitating frailty phenotype.

## Methods

### Subjects.

Twenty-three ambulatory, community-dwelling, older individuals with no history of significant or limiting comorbidities (Supplemental Table 1) were referred to this study from a research registry of older adults. All older subjects referred had their physical frailty status determined using a well-validated aggregate of 5 criteria that includes grip strength, walking speed, weight loss, fatigue, and physical activity measures (1, 56) (Supplemental Table 2). Eleven participants (81 ± 2.7 years, mean ± SEM) met a minimum of 3 or more of 5 physical frailty criteria and were considered frail (FO). Twelve participants (79 ± 2.0 years) comprised a “nonfrail” older group that included 11 robust older adults with a score of 0 and one older adult with a score of 1. When recruiting from the research registry, FO individuals were first enrolled followed by age- and sex-matched NFO individuals to form 2 similar-sized cohorts. Eleven healthy, middle-aged participants (age 51 ± 2.1 years) with no history of diabetes mellitus, hypertension, heart disease, or vascular disease served as CONT and in part were previously reported (57). Individuals were excluded if they were unable to ambulate or exercise, unable to lie flat or complete the MR study, or had implanted devices (i.e., pacemakers, defibrillators) or other hardware contraindicated for MRI. Older participants had the choice to complete all of the studies on the same day or within 1 week.

### Study protocol.

Subjects underwent ^31^P-MRS at rest, dynamic ^31^P-MRS during graded multistage PFE to exhaustion, and during postexercise recovery for assessment of SM energetics in a 3T MRI system (Philips Healthcare) using previously described methods (57). Subjects were seated with their shoulders on a 25.5 cm (10 inch) incline during image acquisition and exercise. The dominant foot was secured to a custom-built MR-compatible plantar-flexion weighted foot pedal to minimize exercise contributions from other muscles. The calf muscles (gastrocnemius, soleus) were centered on a custom-built ^31^P-MRS coil. Position of the calf over the MRI coil was confirmed by scout MRI. Under resting conditions, all participants underwent conventional MRI of the lower extremity for baseline evaluation of calf muscle area and fat content. Muscle composition was ascertained using spin-spin relaxation T2-weighted MRI imaging, in which fat fraction results were measured. As previously described, T2-weighted images (field of view = 220 × 220 × 175 mm^3^, resolution = 0.78 × 0.78 × 10 mm^3^, 16 slices, pulse repetition time = 2141 ms, echo time = 100 ms) were acquired before and after PFE (57). Popliteal artery blood flow was also measured before exercise in older participants using MRI velocity mapping (58).

Prior to exercise, resting-state SM ^31^P-MRS was performed to measure (a) the absolute HEP and Pi concentrations and the unidirectional ATP synthesis rates through (b) the creatine-kinase (CK) reaction using the TRiST method (59) and (c) the ATP→Pi reaction, as previously described (57). After baseline measures, PFE was initiated and plantar flexion was performed every second on a foot pedal connected by a pulley to a weight that was increased at the start of each 120-second exercise stage, as previously described (57). Participants were coached before and during PFE by a research nurse present in the MRI scanner room, and the foot pedal excursion was noted. One-second time cues for plantar flexion were provided by a metronome sound. The first stage commenced with a 0.9 kg weight. A 0.9 kg weight was added at the second stage, and 1.8 kg was added at each subsequent stage. Dynamic ^31^P-MRS data were acquired every 2 seconds, starting 120 seconds before PFE (baseline), continuing throughout exercise and postexercise recovery. During PFE, noting displacement of the weight once per second, total work was calculated (in joules) by the sum of energy for each stage, which was calculated by force (kg × m/s^2^) × distance (m), (i.e., force [weight_pounds_ × 0.453 kg/lbs. × 9.81 m/s^2^] × distance (distance_inches_ × 2.54 cm/in ÷ 100 cm). Exercise was terminated when subjects said they were unable to continue exercise at the prescribed rate of once per second.

Heart rate and blood pressure were measured at each exercise stage using a fingertip pulse oximeter or ECG device, and an automated blood pressure cuff. Subjective fatigue was recorded during each exercise stage using an 11-point (0–10) BORG rating scale of perceived exertion for both leg and total body fatigue (60).

Participants also underwent functional assessments of exercise tolerance using 6MW and bicycle cardiopulmonary stress tests. Supervised 6MW testing commenced after a quiet 10-minute resting period in a 60-foot section of a level hallway free of pedestrian traffic, with clear markers signifying the beginning and end. Distance walked and BORG symptoms were noted at the completion of 6 minutes. (CPET was performed with gas-exchange analysis using a standard cycle ergometer protocol with a 25-watt graded intensity increase every 3 minutes. Subjects exercised to peak fatigue with a target RER >1.1 and a rated perceived exertion >18 for adequate effort. Vital signs, ECG, and BORG symptoms were monitored at each exercise stage and throughout recovery. Oxygen consumption was measured with each breath and averaged over 15-second intervals. Peak oxygen consumption was taken as the average of the 2 highest values of oxygen uptake during the last minute of exercise (57). The 6MW was performed on all participants, except 1 healthy volunteer. Peak VO_2_ during CPET was measured in all participants except 1 frail individual and 1 healthy volunteer.

### Image analysis.

T2-weighted fat images of central slices of calf muscles were processed using Matlab (Mathworks) and segmented manually to remove subcutaneous fat and bones. To compare images among the subject groups, T2-weighted images were normalized by subcutaneous fat signal intensity as previously described (57). Popliteal blood flow was calculated from cine images from the same segment of vessel through each phase of the cardiac cycle using Matlab software.

Absolute concentrations (μmol/g wet weight) were measured using a previously validated external reference method (61). The unidirectional rate of ATP synthesis from both the creatine kinase reaction (PCr to ATP) and from inorganic phosphate (Pi to ATP), were obtained by measuring the PCr or Pi MRS signals in spectra acquired with γ-ATP saturated relative to a control saturation scan, as detailed previously (57, 59). Spectra acquired during PFE were analyzed using the AMARES tool of the jMRUI software package (62). HEPs during exercise were obtained by averaging the last 10 spectra at rest and during each subsequent exercise stage before fitting in AMARES. Cytosolic adenosine diphosphate (ADP) concentration was calculated assuming that 15% of the total creatine was unphosphorylated at rest and an equilibrium constant of K_eq_ = 1.66 × 10^9^ (63, 64). Gibbs free energy was then calculated using the cytosolic ADP, Pi, and ATP concentrations (57, 65). The individual postexercise recovery time for PCr was determined by fitting a mono-exponential function to postexercise PCr after the patient reported exhaustion and stopped exercising (57, 66, 67). Mitochondrial function, as estimated by maximal oxidative capacity, was calculated using Michaelis-Menten kinetics, as previously described (57, 68).

### Statistics.

The Shapiro-Wilk test was used to test whether data were normally distributed. One-way ANOVA corrected for multiple comparisons was used to test for differences among the 3 cohorts in normally distributed variables. The Kruskal-Wallis test corrected for multiple comparisons, and Mann-Whitney 2-tailed pairwise testing was used to test group differences in nonnormally distributed data. Spearman’s correlation tests were used to calculate correlation coefficients. A *P* value less than 0.05 was considered significant. Statistical analysis was performed using Prism version 8 for Windows (GraphPad Software).

### Study approval.

The Johns Hopkins Institutional Review Board approved this study and protocols involving human subjects. All participants were given a detailed explanation of the study protocol, all questions were answered, and each provided informed, written consent before enrollment.

## Author contributions

MS, GG, JDW, and RGW designed the study. MS wrote the MRS acquisition software. SCL, KW, MS, and YZ collected and analyzed the MRS and the MRI data. SCL and TJS performed blinded analyses to test reproducibility of MRS. SCL and QX performed the statistical analysis. JDW referred participants. SCL, AS, and RGW supervised exercise tests. SCL and RGW drafted the manuscript. SCL, PAB, JDW, GG, and RGW edited the manuscript.

## Supplementary Material

supplemental data

ICMJE disclosure forms

## Figures and Tables

**Table 1:**
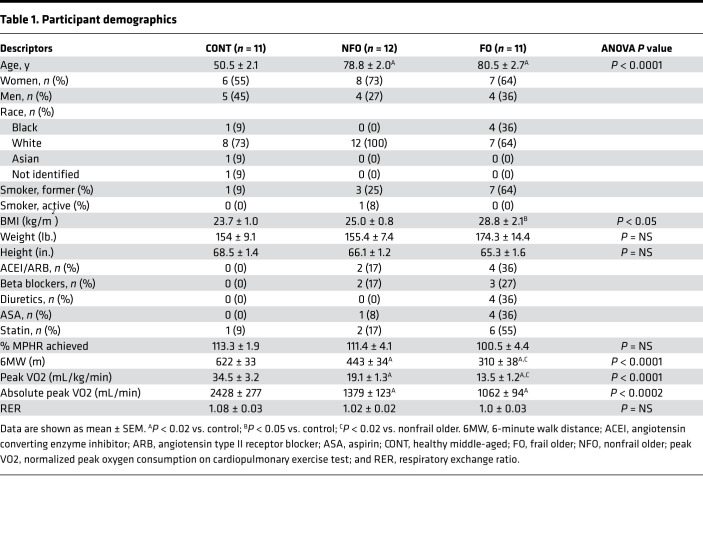
Participant demographics

## Version 1 09/17/2020

In-Press Preview

## Version 2 10/15/2020

Electronic publication
