# Supplementary material for: Exercise intolerance and rapid skeletal muscle energetic decline in human age-associated frailty
Source: JCI Insight. 2020 Oct 15;5(20):e141246. doi: 10.1172/jci.insight.141246 (PMC7605538; doi:10.1172/jci.insight.141246)
Supplement: supplemental data [file jciinsight-5-141246-s256.pdf]

Supplemental Materials

Exercise intolerance and skeletal muscle energetics in human age-associated frailty

Sabra C. Lewsey, MD, MPH[1], Kilian Weiss, PhD[1-3], Michael Schär, PhD[2], Yi Zhang, PhD[2, 4], Paul A. Bottomley, PhD[2], T. Jake Samuel,PhD[1] Qian-Li Xue, PhD[5] Angela Steinberg, MS[1], Jeremy D. Walston, MD[5], Gary Gerstenblith, MD[1], Robert G. Weiss, MD[1]

Affiliations

[1]Division of Cardiology, Department of Medicine, Johns Hopkins University School of Medicine, Baltimore, MD

[2]Division of Magnetic Resonance Research, Department of Radiology, Johns Hopkins University School of Medicine, Baltimore, MD

[3]Philips Healthcare Germany, Hamburg, Germany

[4]Key Laboratory for Biomedical Engineering of Ministry of Education, Department of Biomedical Engineering, College of Biomedical Engineering & Instrument Science, Zhejiang University, Hangzhou, Zhejiang, China

[5]Divison of Geriatric Medicine and Gerontology, Department of Medicine, Johns Hopkins University School of Medicine, Baltimore, MD

Word count:2573

Address for correspondence:

Robert G. Weiss, MD
Blalock 544, Johns Hopkins Hospital
600 N. Wolfe St.
Baltimore, MD 21287-6568
Phone: 410-955-1703
Email: rweiss@jhmi.edu

SUPPLEMENTAL MATERIALS

Table 1: Demographics and co-morbidities of study participants. Ɨp<0.05 vs Control; δ p<0.02 vs Control.

Health Status Descriptors	Healthy Middle-Aged (CONT) (n=11)	Non-Frail Older (NFO) (n=12)	Frail Older (FO) (n=11)	ANOVA p value
Age, y	50.5±2.1	78.8±2.0 δ	80.5±2.7 δ	p < 0.0001
Smoker, former (%)	1 (9)	3 (25)	7 (64)	
Smoker, active (%)	0 (0)	1 (8)	0 (0)	
Body Mass Index (kg/m^2)	23.7 ± 1.0	25.0±0.8	28.8±2.1 Ɨ	p < 0.05
Weight (lb.)	154 ± 9.1	155.4±7.4	174.3±14.4	p = ns
Height (in.)	68.5 ± 1.4	66.1± 1.2	65.3±1.6	p = ns
Medications, n (%)				
ACE-inhibitor/Angiotensin Receptor Blocker	0 (0)	2 (17)	4 (36)	
Beta-Blockers	0 (0)	2 (17)	3 (27)	
Calcium Channel Blockers	0 (0)	0 (0)	2 (18)	
Diuretics (Thiazides/Loop)	0 (0)	0 (0)	4 (36)	
Aspirin	0 (0)	1 (8)	4 (36)	
Statin or Anti-lipidemic	1 (9)	2 (17)	6 (55)	
Oral Hypoglycemic or Insulin	0 (0)	0 (0)	1 (9)	
Oral Steroids	0 (0)	0 (0)	0 (0)	
Co-morbidities, n (%)				
Diabetes Mellitus	0 (0)	0 (0)	2 (18)	
Hypertension	0 (0)	6 (50)	7 (64)	
Hyperlipidemia	1 (9)	6 (50)	7 (64)	
Coronary Artery Disease	0 (0)	1 (8)	1 (9)	
Heart Failure	0 (0)	0 (0)	0 (0)	
Renal Failure	0 (0)	0 (0)	1 (9)	

Table 2: Criteria used to define the frailty phenotype. Frailty was determined if three or more criteria were present based on the metrics determined and reproduced from the Cardiovascular Health Study and Women's Health and Aging Studies[1,2].

Criteria	Description<br>Meets criteria for frailty if ≥ 3 Points:	Points
Weight Loss	Lost > 5% body weight unintentionally in last year, or BMI < 18.5 kg/m^2.	1
Exhaustion	Felt unusually tired or unusually weak 'all of the time' or 'most of the time' or reported energy level was ≤ 3 on a five-part questionnaire.	1
Slowness	Walking speed over 4-meters:<br>Men: ≤0.65 m/s for height ≤ 173 cm (68 inches); ≤0.76 m/s for height >173 cm (68 inches)<br>Women: ≤0.65 m/s for height ≤159 cm (63 inches); ≤0.76 m/s for height >159 cm (63 inches)	1
Low Activity Level	Men:<128 kcal of physical expenditure on activity scale per week (6 items)<br>Women:<90 kcal of physical expenditure on activity scale per week (6 items)	1
Weakness	Grip strength score (hand dynamometer) with dominant hand is:<br>Men: ≤29 kg for BMI ≤24; ≤30 kg for BMI 24.1-26; ≤30 kg for BMI 26.1-28; ≤32 kg for BMI >28<br>Women: ≤17 kg for BMI ≤23; ≤17.3 kg for BMI 23.1-26; ≤18 kg for BMI 26.1-29; ≤21 kg for BMI > 29	1

Figure 1: Relationship between body mass index and calf muscle fat fraction (A) with and (B) without muscle fat fraction outliers. Muscle fat fraction was considered an outlier if intramuscular fat content exceeded 42%, which included two participants (one frail, one non-frail). CONT (n=10, black triangles), NFO (n=12, dark gray circles), FO (n=11, open squares) in plots including outliers, CONT (n=10), NFO (n=11), FO (n=10) in plots without outliers. Statistical tests used were Spearman correlations.

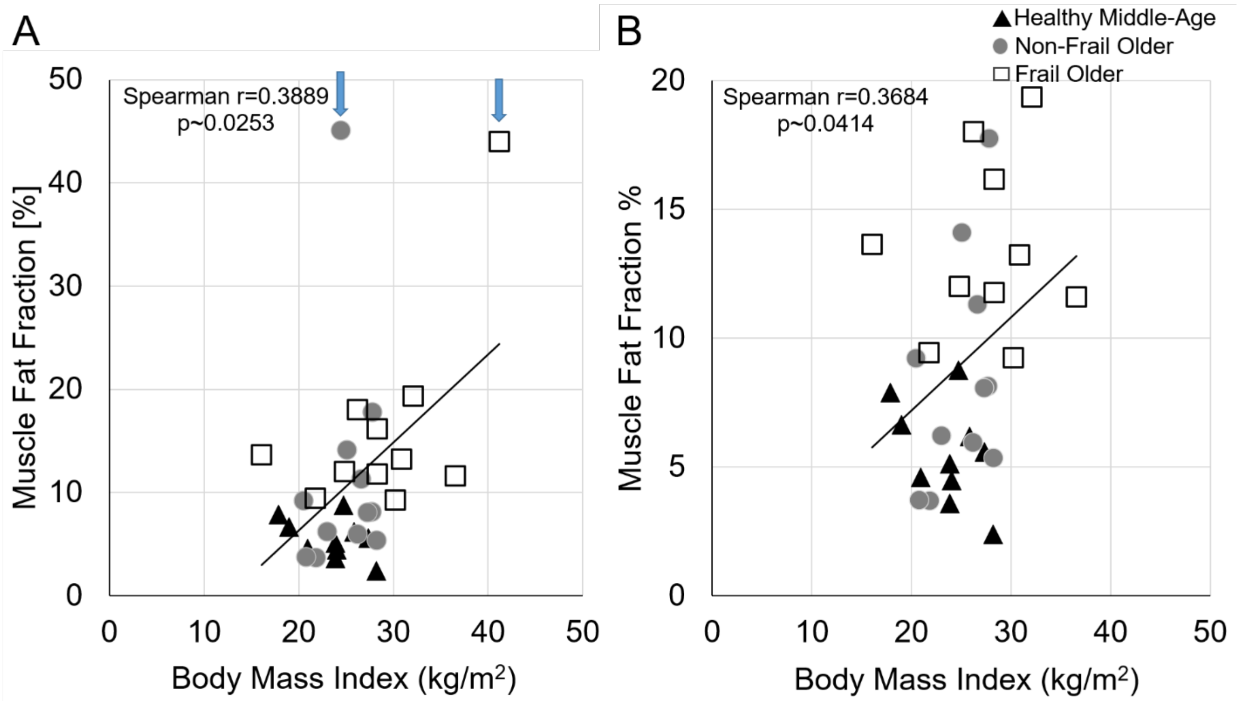

Figure 2: From Figure 6D: PFE time versus muscle fat fraction (A) and without outliers (blue arrows) (B). CONT (n=10, black triangles), NFO (n=12, dark gray circles), FO (n=11, open squares) in plots including outliers, CONT (n=10), NFO (n=11), FO (n=10) in plots without outliers. Statistical tests used were Spearman correlations.

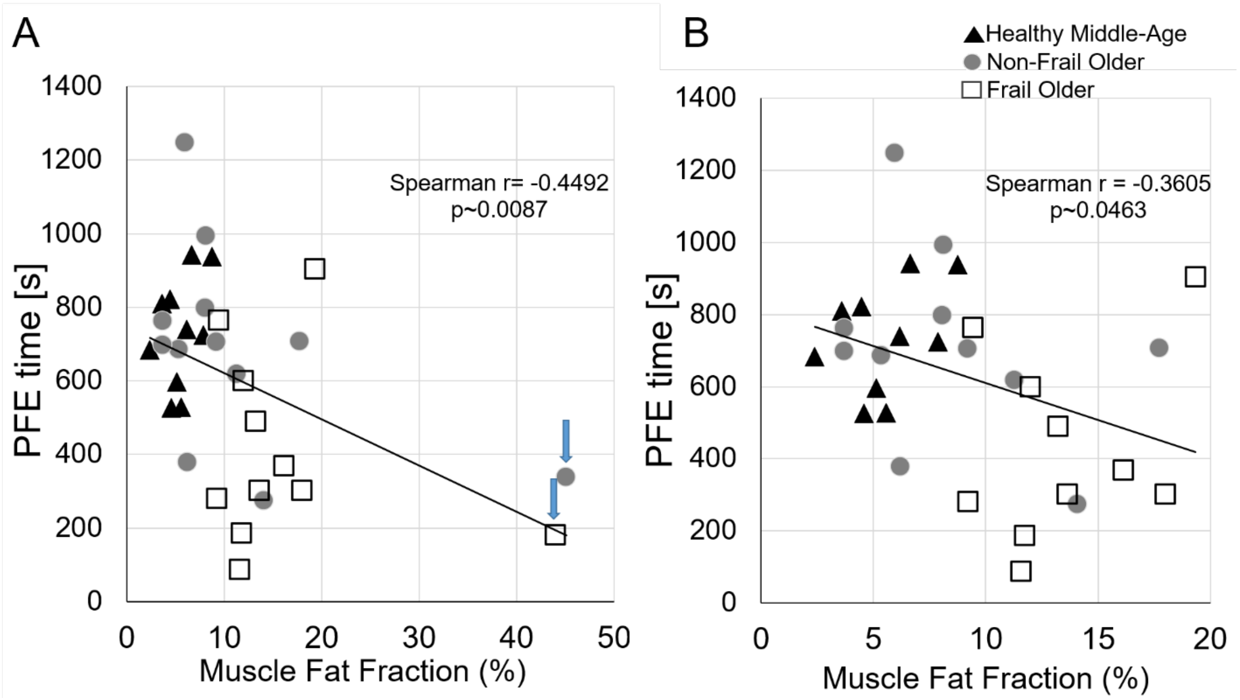

Figure 3: Relationship between body mass index and PFE duration (p=0.0916). CONT (n=11, black triangles), NFO (n=12, dark gray circles), FO (n=11, open squares). Statistical test used was Spearman correlation.

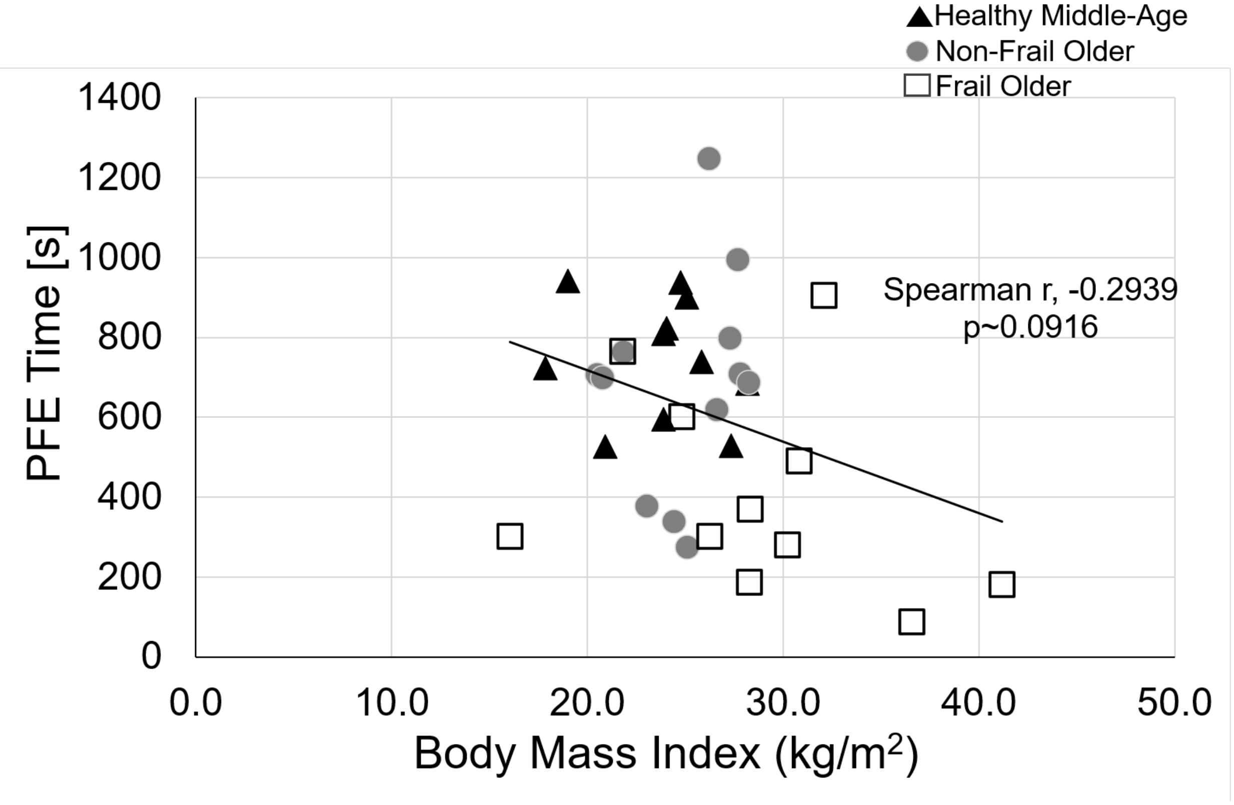

Figure 4: From Figure 6E: Average PCr decline versus muscle fat fraction (A) and without outliers (blue arrows) (B). CONT (n=10, black triangles), NFO (n=12, dark gray circles), FO (n=11, open squares) in plots including outliers, CONT (n=10), NFO (n=11), FO (n=10) in plots without outliers. Statistical tests used were Spearman correlations.

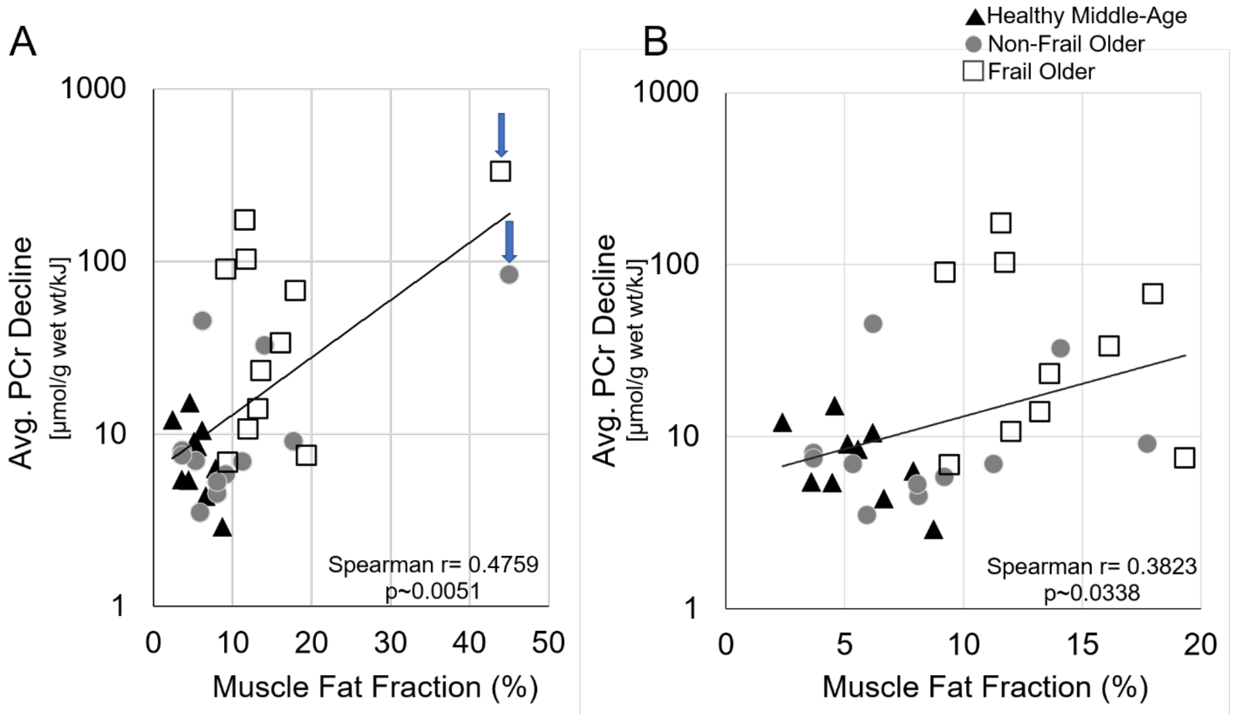

Figure 5: From Figure 6F: Maximal oxidative capacity is inversely related to intramuscular fat fraction (A) and with outliers (blue arrows) removed (B). CONT (n=10, black triangles), NFO (n=12, dark gray circles), FO (n=11, open squares) in plots including outliers, CONT (n=10), NFO (n=11), FO (n=10) in plots without outliers.

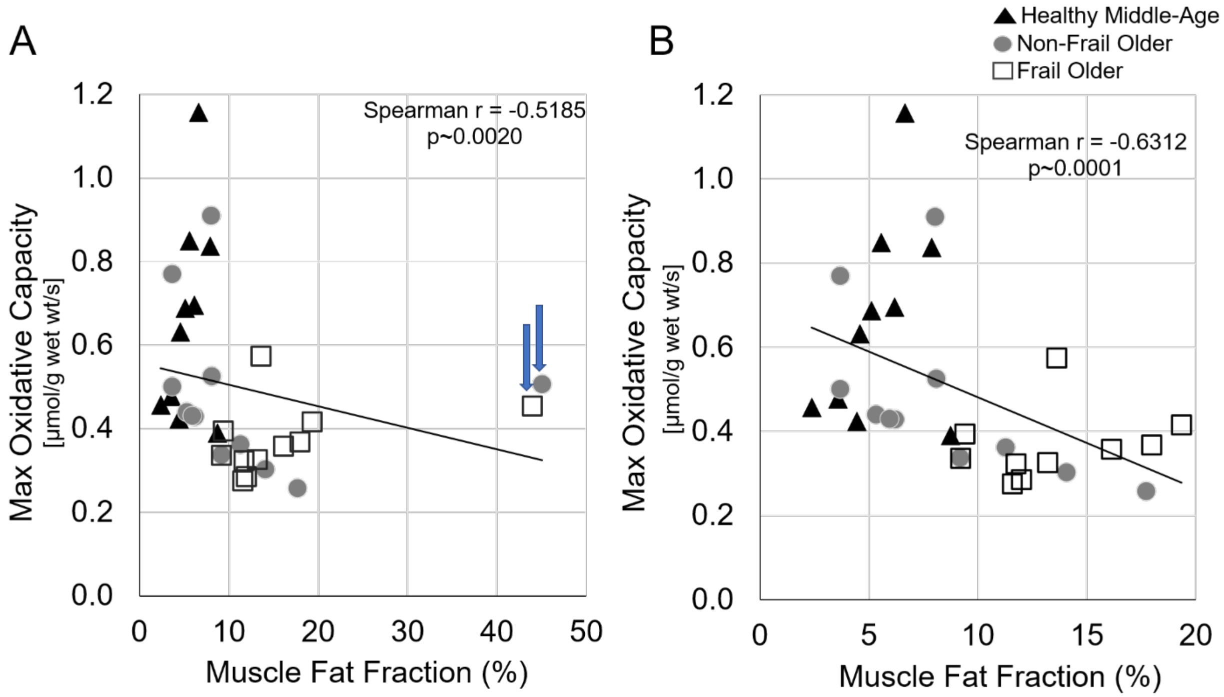

Statistical tests used were Spearman correlations.

Figure 6: Popliteal artery peak blood flow in older individuals at rest, NFO (n=12, dark gray circles), FO (n=11, open squares). Statistical tests used were Mann-Whitney U (two-tailed) tests. A p-value less than 0.05 was considered significant.

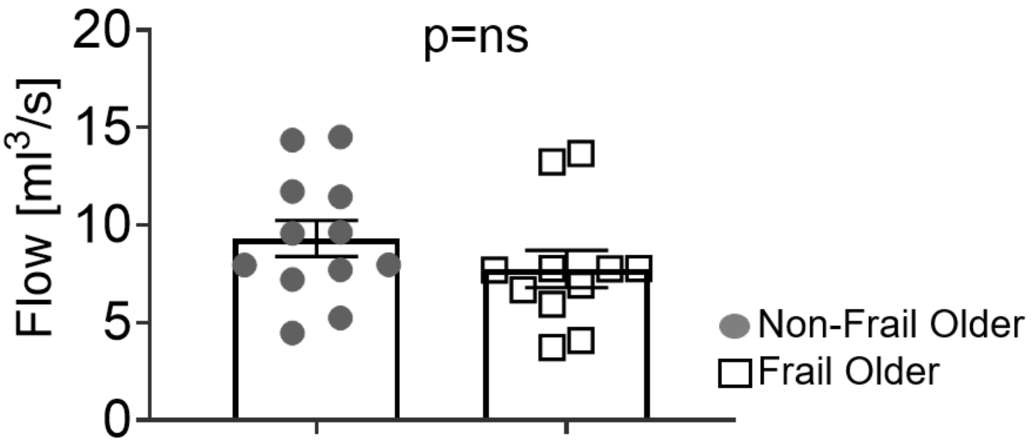

Figure 7: PCr recovery time is directly related to intramuscular fat fraction (%) (A) and with outliers (blue arrows) removed (B). CONT (n=10, black triangles), NFO (n=12, dark gray circles), FO (n=11, open squares) in plots including outliers, CONT (n=10), NFO (n=11), FO (n=10) in plots without outliers. Statistical tests used were Spearman correlations.

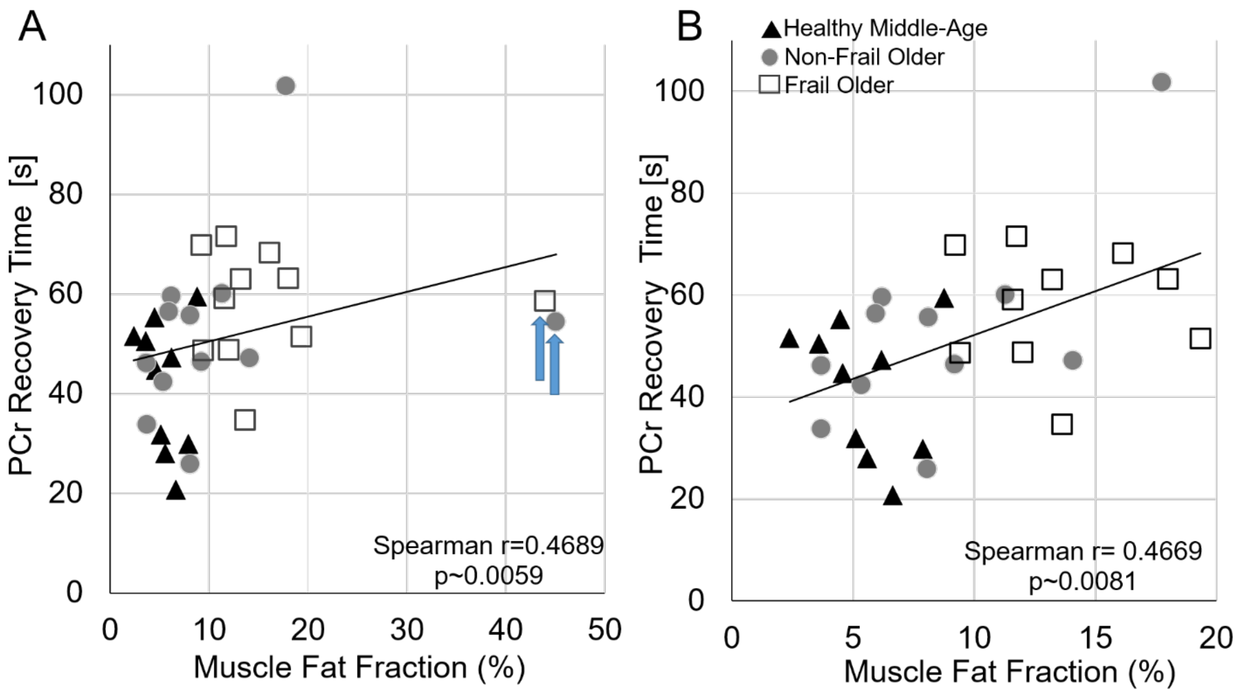

Figure 8: Walking speed during clinical frailty assessment is directly correlated with rate of energetic decline. NFO (n=12, dark gray circles), FO (n=11, open squares). Statistical tests used were Spearman correlations.

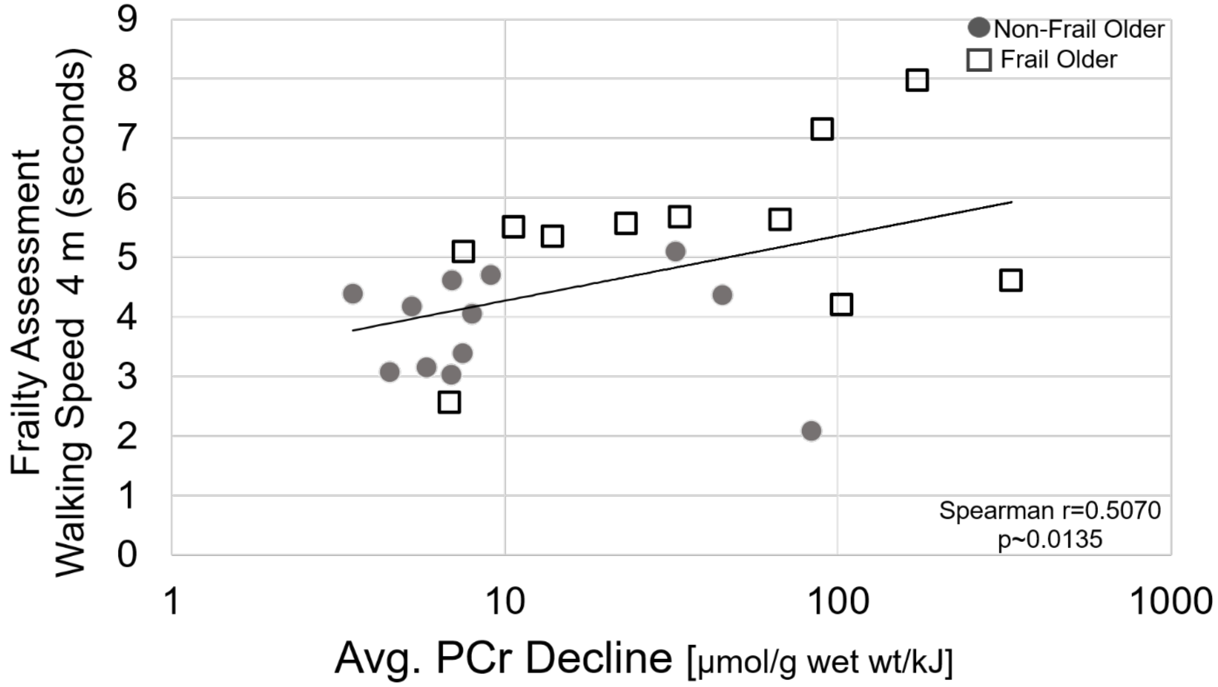

Figure 9: Phosphocreatine decline per minute in the first two minutes of exercise, not normalized to work performed. During the first two minutes of exercise, all participants lifted two pounds during plantar flextion. Frail older individuals exhibit several fold-faster rates of phosphocreatine decline. CONT (n=11, black triangles), NFO (n=12, dark gray circles), FO (n=11, open squares). Data are means±SEM. Statistical tests were Kruskal-Wallis ANOVA with Mann-Whitney U tests. * $p < 0.05$, ** $p < 0.02$, *** $p < 0.005$, **** $p < 0.001$.

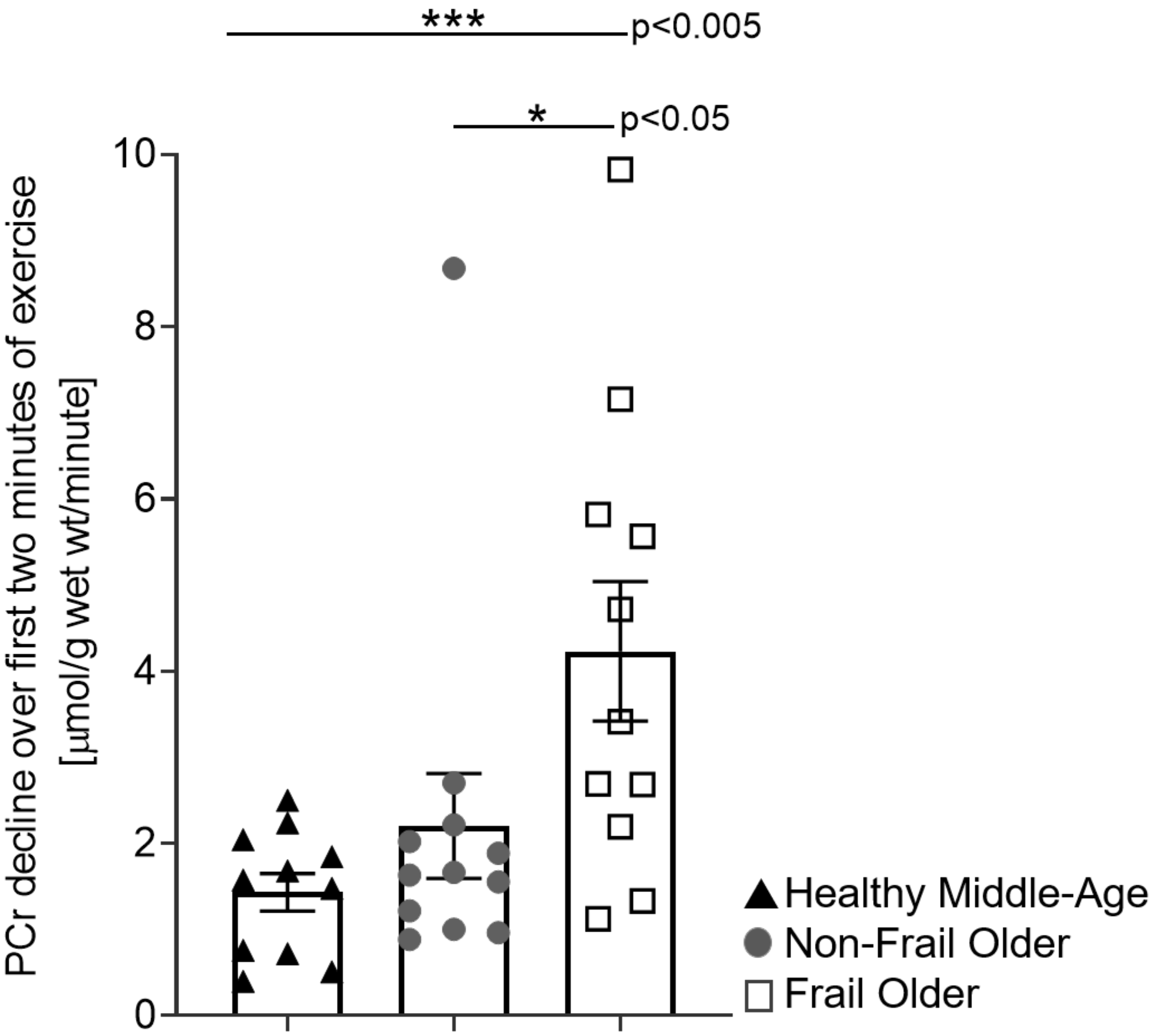

Figure 10: Reliability and reproducibility of magnetic resonance spectroscopy analysis methods. MR analysis performed by two different investigators blinded to each other's results. (A-C) Intraclass correlations, (D-F) Bland-Altman plots, (G) Table of intraclass correlation coefficients (ICC) of energetic parameters.

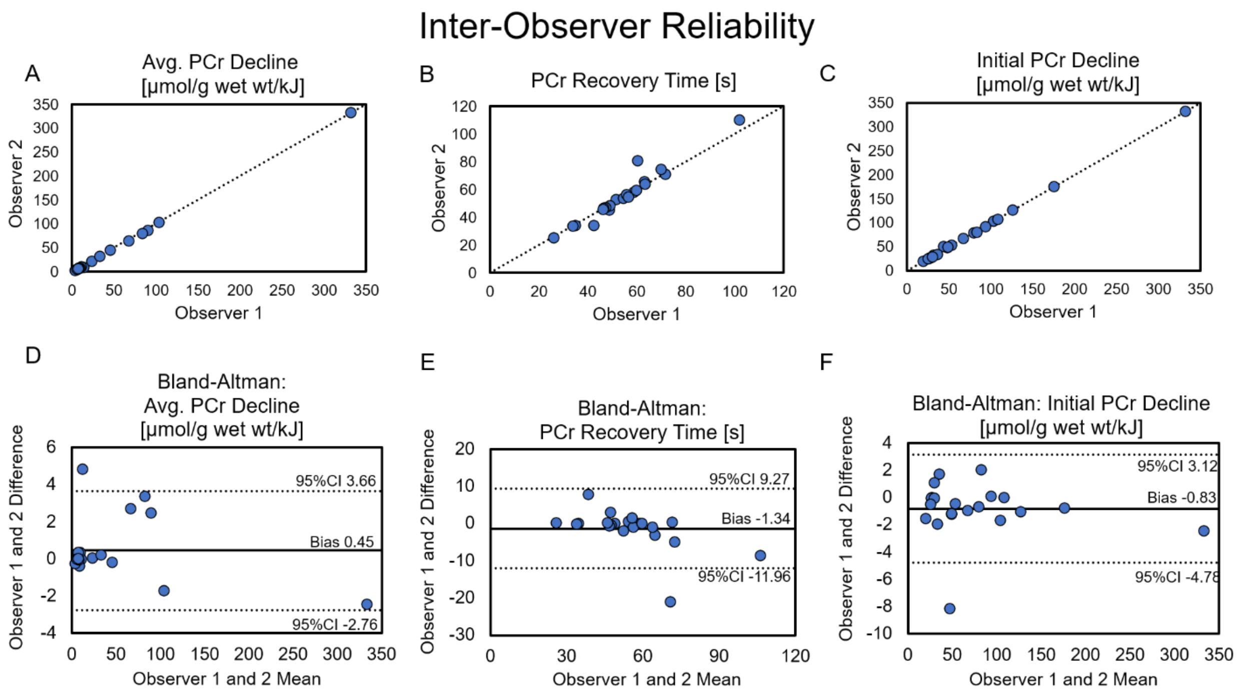

G

Energetic Parameter	CV [%]	ICC
Avg. PCr Decline [µmol/g wet wt/kJ]	2.8	0.99
Initial PCr Decline [µmol/g wet wt/kJ]	2.0	0.99
VmaxPCr [µmol/g wet wt/s]	7.8	0.96
PCr Recovery [s]	7.0	0.95

2. Supplemental Methods

Health Status and Frailty Assessment:

Health status (Table 1) was obtained by assessment of past medical history, smoking status, active medications, and review of health records.

Criteria used to define the frailty phenotype (Table 2) were taken from the Cardiovascular Health Study and Women's Health and Aging Studies[1,2]. From the Johns Hopkins Older Americans Independence Center, physical activity was based on modified Minnesota Leisure Time Activities Questionnaire[3]. Activity inquiries included asking about walking (*w*=3.5), strenuous household chores (*w*= 4.5), strenuous outdoor chores (*w*= 4.5), dancing (*w*= 5.5), bowling (*w*= 3.0), and exercise (*w*=4.5).

To compute kcals expended per week, where *w* is the task-specific (metabolic equivalent task) MET intensity score, F is the frequency of sessions per week, D is the duration of the session in minutes, and B is body weight in kilograms, we used the formula:

Kcals (kilocalories/week) = $w^* F^* D^* (B/60)$.

The equipment to assess weight loss included a scale for body weight and stadiometer for height. Walking speed was assessed averaging two trials of the participant walking his or her usual pace twice on a 4-meter course supervised and timed with a stopwatch. Weakness was assessed by the participant squeezing a dynamometer maximally three times with their dominant hand.

Study Design:

Informed consent after explanation of the study protocol was obtained from all participants. The Johns Hopkins Institutional Review Board (IRB) approved all human studies (Baltimore, MD). ^{31}P MRI/MRS data were collected prospectively and analyzed by investigators (SCL, KW, MS, YZ, and TJS) blinded to clinical status (6MW, peak-VO_2 performance).

1H MRI image acquisition and analysis:

All ^1H MRI examinations were performed with the body coil in transmit and receive mode. Muscle composition of the calf muscle was estimated with a set of spin-spin relaxation (T2) weighted images acquired before PFE. Matlab (Mathworks, Natick MA) was used for image processing. In order to measure fat content of the skeletal muscle images, a central T2-weighted image was segmented manually and intramuscular fat fractions were calculated. The signal intensity of the subcutaneous fat layer was used to normalize T2-weighted images. Any pixel values above a threshold of 10% were averaged to estimate fat fraction[4].

31P MRS data analysis:

Quantification of HEP metabolite concentrations was performed through a validated method previously described and concentrations were not assumed[5,6]. The unidirectional rate of ATP synthesis through the creatine kinase reaction (PCr → ATP) and from Pi (Pi → ATP) were calculated by quantifying the magnetization of PCr and Pi respectively in the ɣ-ATP saturated spectra using methodologies previously described[7]. In order to perform the latter, an additional scan with control saturation referenced to the Pi resonance was also acquired. Analysis of dynamic 31 P MRS spectra was performed before, during and after PFE exercise using the AMARES function of jMRUI[8]. The last ten spectra at baseline and during each exercise stage were averaged independently prior to fitting to estimate HEP before and during dynamic exercise. Intracellular pH was quantified by the difference in chemical shift of the Pi and PCr resonances[9]. Using an equilibrium constant of $K_{eq}=1.66x10^9$ and assuming at rest that 15% of total creatine is unphosphorylated, the concentration of cytosolic ADP was calculated using[10,11]:

$$[ADP]= [ATP][Cr] \div [PCr][H+]K_{eq}.$$

PCr decline during exercise was estimated by linear regression of PCr concentration versus the individual work performed at every stage in each subject. Given the range in magnitudes and considerably higher normalized rates of PCr decline in frail older individuals, a logarithmic scale was used to compare average PCr decline with functional indices (PFE time, 6MW distance, peak-VO_2 during CPET).

The energy release of ATP hydrolysis (i.e. Gibbs free energy) was calculated with previously described methods[12], using the standard free energy change (ΔG_0), the universal gas constant (R), and absolute temperature (T), and thus:

$$\Delta G_{ATP}= \Delta G_0+ R * T * \log ([ADP][Pi]/[ATP]).$$

Post-exercise recovery kinetics of PCr were quantified after exercise was terminated after attaining self-reported maximal fatigue by each subject. PCr recovery time (τPCr) for each individual was estimated through the fitting of a mono-exponential function. Mitochondrial function was approximated by maximal oxidative capacity in the rate of PCr repletion per second ($Vmax_{PCr}$). Using Michaelis-Menten kinetics, we estimated $Vmax_{PCr}$ by assuming a Km of 25 µM [13], and utilizing ADP concentration at the end of exercise (ADP_{end}, maximal fatigue) and the initial rate of PCr recovery (Vi_{PCr}), to determine:

$$Vmax_{PCr}=Vi_{PCr} (1 + (Km/[ADP_{end}])).$$

Vi_{PCr} was determined using 1) estimated τPCr, and 2) the difference in concentration of PCr between baseline at rest and at end exercise at fatigue (ΔPCr), thus:

$$Vi_{PCr}= (1/\tau PCr)*\Delta PCr.$$

It is important to note that PCr recovery kinetic analysis is independent of muscle size.

Six Minute walk and Cardiopulmonary exercise testing (CPET):

Six-minute walk tests were performed on an enclosed 60-foot corridor, free of traffic, and with chairs marking each end. Participants were instructed prior to the test to walk at a pace that they chose and to rest if necessary. They sat quietly for 10 minutes before starting the supervised walk. The subjects were observed but not coached as they walked their maximum level distance during six minutes. At the conclusion of six minutes, the distance walked and level of fatigue per the BORG scale were recorded[14]. CPET was performed a minimum of 2 hours after taking medications with gas-exchange analysis on a *MedGraphics Ultima* cart during a cycle ergometer protocol with 25-watt graded increases in intensity every 3 minutes. Heart rate, blood pressure, and an ECG were recorded at each stage and during recovery. BORG symptoms were also recorded during each exercise stage as well as any symptoms associated with the termination of exercise[15-17]. All patients exercised to exhaustion to a goal respiratory exchange ratio (RER) > 1.1 and a rated perceived exertion (RPE) > 18. Breath by breath oxygen consumption was measured and reported in 15 second averaged intervals. Peak venous oxygen consumption was defined as the average of the two highest oxygen uptake values during the last minute of exercise.

Supplemental References:

1. Fried LP, Tangen CM, Walston J, et al. Frailty in older adults: evidence for a phenotype. *J Gerontol A Biol Sci Med Sci.* 2001;56(3):M146-156.
2. Bandeen-Roche K, Xue QL, Ferrucci L, et al. Phenotype of frailty: characterization in the women's health and aging studies. *J Gerontol A Biol Sci Med Sci.* 2006;61(3):262-266.
3. Taylor HL, Jacobs DR, Jr., Schucker B, Knudsen J, Leon AS, Debacker G. A questionnaire for the assessment of leisure time physical activities. *J Chronic Dis.* 1978;31(12):741-755.
4. Weiss K, Schar M, Panjrath GS, et al. Fatigability, Exercise Intolerance, and Abnormal Skeletal Muscle Energetics in Heart Failure. *Circ Heart Fail.* 2017;10(7).
5. El-Sharkawy AM, Schär M, Ouwerkerk R, Weiss RG, Bottomley PA. Quantitative cardiac 31P spectroscopy at 3 Tesla using adiabatic pulses. *Magn Reson Med.* 2009;61(4):785-795.
6. El-Sharkawy A-MM, Gabr RE, Schär M, Weiss RG, Bottomley PA. Quantification of human high-energy phosphate metabolite concentrations at 3 T with partial volume and sensitivity corrections. *NMR Biomed.* 2013;26(11):1363-1371.
7. Schär M, El-Sharkawy AMM, Weiss RG, Bottomley PA. Triple repetition time saturation transfer (TRiST) 31P spectroscopy for measuring human creatine kinase reaction kinetics. *Magn Reson Med.* 2010;63(6):1493-1501.
8. Naressi A, Couturier C, Devos JM, et al. Java-based graphical user interface for the MRUI quantitation package. *MAGMA.* 2001;12(2-3):141-152.
9. Taylor DJ, Bore PJ, Styles P, Gadian DG, Radda GK. Bioenergetics of intact human muscle. A 31P nuclear magnetic resonance study. *Mol Biol Med.* 1983;1(1):77-94.

10. Veech RL, Lawson JW, Cornell NW, Krebs HA. Cytosolic phosphorylation potential. *J Biol Chem.* 1979;254(14):6538-6547.
11. Boska M. ATP production rates as a function of force level in the human gastrocnemius/soleus using 31P MRS. *Magn Reson Med.* 1994;32(1):1-10.
12. Gibbs C. The cytoplasmic phosphorylation potential. Its possible role in the control of myocardial respiration and cardiac contractility. *J Mol Cell Cardiol.* 1985;17(8):727-731.
13. Kemp GJ, Taylor DJ, Thompson CH, et al. Quantitative analysis by 31P magnetic resonance spectroscopy of abnormal mitochondrial oxidation in skeletal muscle during recovery from exercise. *NMR Biomed.* 1993;6(5):302-310.
14. Guyatt GH, Sullivan MJ, Thompson PJ, et al. The 6-minute walk: a new measure of exercise capacity in patients with chronic heart failure. *Can Med Assoc J.* 1985;132(8):919-923.
15. Russell SD, Koshkarian GM, Medinger AE, Carson PE, Higginbotham MB. Lack of effect of increased inspired oxygen concentrations on maximal exercise capacity or ventilation in stable heart failure. *Am J Cardiol.* 1999;84(12):1412-1416.
16. Beere PA, Russell SD, Morey MC, Kitzman DW, Higginbotham MB. Aerobic exercise training can reverse age-related peripheral circulatory changes in healthy older men. *Circulation.* 1999;100(10):1085-1094.
17. Borlaug BA, Melenovsky V, Russell SD, et al. Impaired chronotropic and vasodilator reserves limit exercise capacity in patients with heart failure and a preserved ejection fraction. *Circulation.* 2006;114(20):2138-2147.
